# Omics Studies in Osteoarthritis: A Review of Molecular Mechanisms and Candidate Molecules

**DOI:** 10.3390/biom16091363

**Published:** 2026-09-18

**Authors:** Yimeng Sun, Qulong Xiao, Ming Jin, Ge Liu, Qingzhong Zhang, Xiaowei Wei

**Affiliations:** 1 Zhongshan Clinical College, Dalian University, Dalian 116622, China; sunyimeng1004@163.com (Y.S.); xxr980125@126.com (Q.X.); jaz20240121@163.com (M.J.); 2 Laboratory of Orthopedics, Affiliated Zhongshan Hospital of Dalian University, Dalian 116001, China; liuge@dlu.edu.cn

**Keywords:** knee osteoarthritis (KOA), hip osteoarthritis (HOA), transcriptomics, proteomics, differentially expressed genes (DEGs), differentially expressed proteins (DEPs)

## Abstract

Osteoarthritis (OA) is a joint disease of unknown etiology that can occur in joints throughout the body, especially with articular cartilage lesions of the knee joint and hip joint. Osteoarthritis not only causes joint pain, deformity, and functional impairment, but also significantly increases the risk of cardiovascular events, deep vein thrombosis in the lower limbs, and hip fractures. Up to now, there have been no specific medications available for the treatment of osteoarthritis, and the disease remains incurable. Therefore, identifying reliable biomarkers is crucial for achieving a more accurate diagnosis of OA and for guiding the development of targeted treatment strategies. In this paper, we screen and synthesize articles that performed transcriptomics and proteomics analysis on tissue or cell samples taken from osteoarthritis cartilage. We identify common differentially expressed genes (co-DEGs) and proteins (co-DEPs) in cartilage samples from knee osteoarthritis (KOA) and hip osteoarthritis (HOA) and consolidate them into candidate molecules, while systematically delineating their expression trends and underlying mechanisms at both the transcriptomic and proteomic levels. This review establishes a theoretical foundation and identifies future research directions for functional validation and clinical translation of these candidate molecules as potential biomarkers and therapeutic targets for osteoarthritis.

## 1. Introduction

Osteoarthritis (OA) is a chronic disease primarily characterized by degenerative changes in articular cartilage. Its core features include progressive destruction of articular cartilage, accompanied by abnormal subchondral bone remodeling, synovial cell hyperproliferation, and osteophytes at the joint margin [1,2,3]. It can lead not only to joint pain, deformity, and dysfunction [4,5,6], but also to a significant increase in the risk of cardiovascular events [7,8], lower-extremity deep venous thromboembolism [9], and hip fracture [10,11]. The global prevalence of OA continues to rise, currently affecting approximately 7.6% of the world’s population [12,13]. Statistics indicate that the incidence rate of OA is significantly higher in women than in men [14,15,16]. The incidence rate is extremely high in the elderly, and the dysfunction is severe, with patients in the advanced stage potentially losing their ability to work due to joint deformity and dysfunction [17,18,19]. In addition, the incidence rate of OA is closely related to factors such as obesity, joint injury, trauma, race and economic income [20,21,22,23,24]. Currently, the main diagnostic methods for OA are signs and symptoms and imaging, and there is a lack of mature biomarkers that can be used for routine clinical diagnosis [25,26,27]. Treatment methods are also limited to symptomatic treatment, such as analgesia and anti-inflammatory drugs and surgical treatment in the terminal stage, with no specific therapeutic drugs available [28,29,30]. Therefore, this article aims to identify key molecular targets by systematically reviewing the OA-related transcriptomics and proteomics literature, providing new insights for the diagnosis and treatment of OA.

Knee osteoarthritis (KOA) and hip osteoarthritis (HOA) are the most common types of OA. KOA and HOA share significant similarities: First of all, the core pathological change in both is characterized by progressive degeneration and damage of the articular cartilage, ultimately leading to joint structure destruction and functional impairment [31,32,33]. Secondly, the main clinical manifestations of both tend to be consistent, including symptoms such as joint pain, stiffness, limited mobility, and functional decline [17,21,24,34]. Finally, conservative treatment strategies also follow common principles, including disease education, weight management, exercise therapy, and physical therapy [35,36,37].

Concurrently, there are significant differences between KOA and HOA. First and foremost, regarding anatomical structure, the knee is a hinge joint that allows flexion and extension with rotation and contains a meniscus [38], whereas the hip is a ball-and-socket joint with a greater range of motion and no meniscus [39]. Additionally, Bastick et al. found that malalignment is a significant risk factor for knee osteoarthritis, while its association with HOA is relatively weaker [40]. Therefore, biomechanical strategies to improve symptoms and delay disease progression are one of the core directions in the field of osteoarthritis research [41]. Finally, the location of pain is the key clinical feature distinguishing the two conditions. The pain of knee osteoarthritis is usually located in the anterior knee, medial side, or lateral region and sometimes radiates to the proximal lower leg. Patients experience particularly prominent pain during stair climbing [42]. In contrast, the typical pain location of HOA is in the groin region, followed by the buttock, anterior thigh, or medial side [43]. It is worth noting that hip pain can sometimes radiate to the knee joint, a phenomenon that may lead to misdiagnosis as KOA. Therefore, identifying the differentially expressed genes in KOA and HOA through omics, and then finding targets, are helpful for the diagnosis and treatment of the two.

Transcriptomics is the study of all transcripts in a specific cell, tissue, or organism at a particular time point or under specific conditions [44,45,46]. It encompasses all RNA molecules present in a cell or tissue at a given time, while focusing on quantifying gene expression, understanding gene function, and mapping regulatory networks that drive biological processes [47,48,49]. Early traditional sequencing methods typically targeted a single gene or a small number of genes, employing the Sanger chain-termination method and Maxam–Gilbert chemical degradation method [50,51,52]. Subsequently, the first generation of large-scale transcriptomics analysis technology emerged: the gene expression microarray [47,48]. This technology immobilizes DNA probes of known sequences on a chip, reverse transcribes RNA from samples into cDNA and labels it with fluorescence, and then hybridizes it with the chip. By detecting the intensity and location of fluorescent signals, it determines which genes are expressed and their expression level. However, first-generation sequencing techniques rely on probe design and thus they can only detect known sequences, which limits the sequencing range. Currently, RNA sequencing is the mainstream technology in transcriptomics research, enabling open-ended, whole-transcriptome detection of samples in a single experiment and providing read-based expression quantification [53]. In recent years, single-cell transcriptomics [54] and spatial transcriptomics [55,56,57] have greatly expanded the dimensions and depth of transcriptomics research, making it possible to resolve gene expression at cellular resolution and within a spatial context. With the advances and developments that have occurred in transcriptomics technologies, we expect to gain a more comprehensive understanding of the molecular mechanisms involved in the development and progression of osteoarthritis [58]. This review integrates bulk RNA-seq and single-cell RNA-seq datasets. The former is cost-effective, reliable, and provides broad coverage, making it suitable for large clinical cohorts; however, it masks cellular heterogeneity and fails to identify rare cell types. Although the latter can resolve rare cells and developmental trajectories, it is costly, poorly scalable, and subject to experimental biases. To address this, we performed a joint analysis of cartilage tissue data and chondrocyte data.

Proteomics enables the systematic investigation of all proteins expressed in a specific cell, tissue, or organism under defined conditions [59,60,61]. Protein composition within an organism varies significantly depending on cell type, developmental stage, environmental conditions, and physiological state. Therefore, proteomics can more directly reflect the actual functional state of an organism [62,63]. Proteomics requires a combination of various techniques: two-dimensional gel electrophoresis (2-DE) and multiple mass spectrometry techniques [64,65]. 2-DE separates complex protein mixtures based on isoelectric point and molecular weight, generating individual protein spot maps [66,67]. This supports large-scale protein expression pattern analysis and reveals dynamic changes under various biological conditions. 2-DE is particularly suitable for differential proteome analysis and post-translational modification studies [68,69]. However, it also has limitations, such as low throughput, low sensitivity, and poor reproducibility. These shortcomings have largely limited in-depth proteomics research [70,71,72]. Currently, many proteomics studies have shifted to liquid chromatography–tandem mass spectrometry (LC-MS/MS)-based techniques, which offer higher throughput, a wider dynamic range, better sensitivity, and greater automation [73,74,75]. These features make LC-MS/MS more suitable for analyzing complex samples and low-abundance proteins [76,77,78]. The relatively high throughput of this technology also makes it suitable for clinical applications involving human biological fluids and diseased tissues [79]. In recent years, single-cell proteomics has also emerged, enabling direct readout of the functional state of individual cells [80,81,82,83]. However, the limited number of proteins in cells poses significant challenges for mass spectrometry analysis [84]. Advances in proteomics technologies have created opportunities for the discovery of biomarkers for osteoarthritis [85].

This study systematically screened for differentially expressed factors (DEFs) shared at the omics level between cartilage tissues or chondrocytes of KOA and HOA. However, the conclusions are limited to exploratory evidence and have not yet addressed key aspects such as diagnostic performance evaluation, accessibility validation in peripheral biological samples, temporal dynamic associations, causal perturbation analysis, drug safety assessment, or translational feasibility for drug development. Therefore, all differentially expressed factors identified in this study are cautiously defined as “candidate molecules” rather than validated biomarkers or therapeutic targets. More advanced validation work awaits further investigation in subsequent studies.

## 2. Methods

We conducted a literature search in the PubMed database on 30 November 2025 for original research on the transcriptomics and proteomics of osteoarthritis. The search included the following terms: cartilage, osteoarthritis (OA), knee, hip, transcriptome, proteome, RNA sequencing (RNA-Seq), gene expression, and mass spectrometry (MS). This study followed the PRISMA framework for literature search, screening, and structured data extraction. The search strategy was as follows: (Full search details are available in Table S1).

①((((cartilage[Title/Abstract]) AND ((osteoarthritis[Title/Abstract]) OR (OA[Title/Abstract]))) AND (knee[Title/Abstract]) AND(hip[Title/Abstract]))) AND ((transcriptom*[Title/Abstract]) OR (RNA sequencing[Title/Abstract]) OR (RNA-Seq[Title/Abstract]) OR (Gene expression[Title/Abstract]))

②((((cartilage[Title/Abstract]) AND ((osteoarthritis[Title/Abstract]) OR (OA[Title/Abstract]))) AND (knee[Title/Abstract]) AND(hip[Title/Abstract]))) AND ((proteom*[Title/Abstract]) OR (Mass spectrometry[Title/Abstract]) OR (MS[Title/Abstract]) OR (Molecular profiling[Title/Abstract]))

③(((cartilage[Title/Abstract]) AND ((osteoarthritis[Title/Abstract]) OR (OA[Title/Abstract]))) AND (knee[Title/Abstract])) AND ((transcriptom*[Title/Abstract]) OR (RNA sequencing[Title/Abstract]) OR (RNA-Seq[Title/Abstract]) OR (Gene expression[Title/Abstract]))

④(((cartilage[Title/Abstract]) AND ((osteoarthritis[Title/Abstract]) OR (OA[Title/Abstract]))) AND (knee[Title/Abstract])) AND ((proteom*[Title/Abstract]) OR (Mass spectrometry[Title/Abstract]) OR (MS[Title/Abstract]) OR (Molecular profiling[Title/Abstract]))

⑤(((cartilage[Title/Abstract]) AND ((osteoarthritis[Title/Abstract]) OR (OA[Title/Abstract]))) AND (hip[Title/Abstract])) AND ((transcriptom*[Title/Abstract]) OR (RNA sequencing[Title/Abstract]) OR (RNA-Seq[Title/Abstract]) OR (Gene expression[Title/Abstract]))

⑥(((cartilage[Title/Abstract]) AND ((osteoarthritis[Title/Abstract]) OR (OA[Title/Abstract]))) AND (hip[Title/Abstract])) AND ((transcriptom*[Title/Abstract]) OR (Mass spectrometry[Title/Abstract]) OR (MS[Title/Abstract]) OR (Molecular profiling[Title/Abstract]))

### 2.1. Inclusion Criteria

Inclusion criteria for search strategies ① and ②—for Section 4, “Common differentially expressed factors (Co-DEFs) in cartilage samples from KOA and HOA patients”: (1) Original studies that performed transcriptomic or proteomic analyses on both knee osteoarthritis and hip osteoarthritis; (2) samples obtained from cartilage tissue or chondrocytes; (3) all species with osteoarthritis; and (4) full-text research in English

Inclusion criteria for search strategies ③ and ④—for Section 5, “Multi-omics characteristic molecules of KOA cartilage”: (1) Original research on transcriptomic or proteomic analysis of knee osteoarthritis; (2) samples obtained from cartilage tissue or chondrocytes; (3) all species with osteoarthritis; and (4) full-text research in English

Inclusion criteria for search strategies ⑤ and ⑥—for Section 6, “Multi-omics characteristic molecules of HOA cartilage”: (1) Original research on transcriptomic or proteomic analysis of hip osteoarthritis; (2) samples obtained from cartilage tissue or chondrocytes; (3) all species with osteoarthritis; and (4) full-text research in English

### 2.2. Exclusion Criteria

Common exclusion criteria for all search strategies: (1) Non-OA omics original research (e.g., reviews, retrospective studies, bibliometric analyses); (2) articles without relevant differentially expressed genes; (3) articles with samples obtained from non-cartilage tissues or chondrocytes (e.g., meniscus, subchondral bone, synovium, plasma); and (4) articles with full text unavailable or only an abstract available.

Exclusion criteria specific to search strategies ① and ②—for Section 4, “Common differentially expressed factors (Co-DEFs) in cartilage samples from KOA and HOA patients”: Original transcriptomic or proteomic research that did not conduct a combined analysis of knee and hip osteoarthritis.

Exclusion criteria specific to search strategies ③ and ④—for Section 5, “Multi-omics characteristic molecules of KOA cartilage”: No original transcriptomic or proteomic research has been conducted on knee osteoarthritis.

Exclusion criteria specific to search strategies ⑤ and ⑥—for Section 6, “Multi-omics characteristic molecules of HOA cartilage”: No original transcriptomic or proteomic research has been conducted on hip osteoarthritis.

## 3. Result

### 3.1. Results of Literature Search

**Figure 1:**
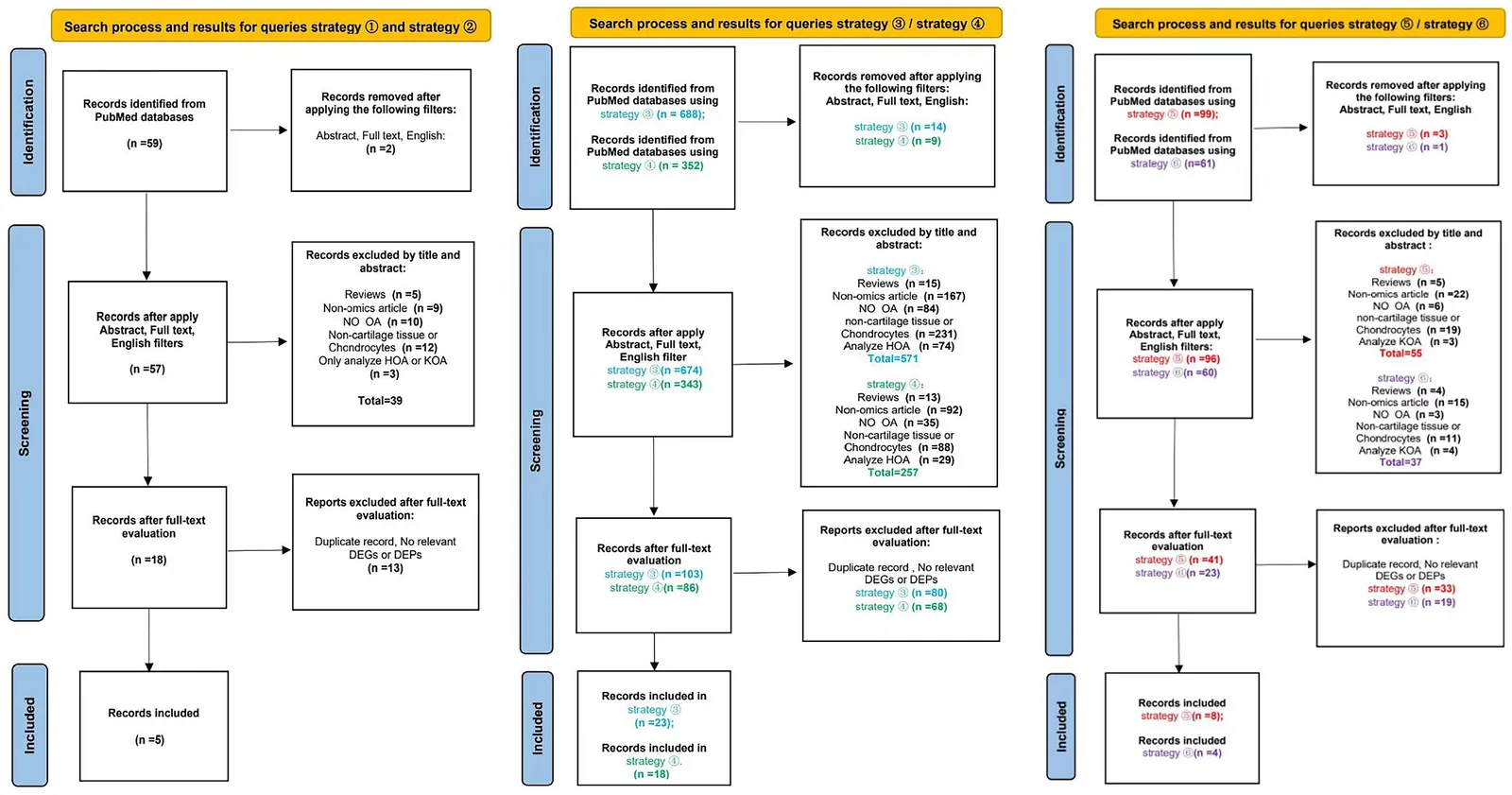
Literature screening flow diagram.

The PubMed search generated 1259 records. Subsequently, we applied the following screening criteria: abstracts, full texts, and English language as inclusion criteria. Search strategies ① and ② together excluded two records; search strategy ③ excluded 14 records; search strategy ④ excluded nine records; search strategy ⑤ excluded three records; and search strategy ⑥ excluded one record, resulting in a total of 29 excluded records. Next, based on the remaining inclusion and exclusion criteria, screening was performed by title and abstract: search strategies ① and ② together excluded 39 records; search strategy ③ excluded 571 records; search strategy ④ excluded 257 records; search strategy ⑤ excluded 55 records; and search strategy ⑥ excluded 37 records, yielding a total of 959 excluded records. Finally, the remaining 271 records were imported into Zetero software (6.0.23), and we performed deduplication and eligibility assessment on the full texts of these records: search strategies ① and ② together excluded 13 records; search strategy ③ excluded 80 records; search strategy ④ excluded 68 records; search strategy ⑤ excluded 33 records; and search strategy ⑥ excluded 19 records, totaling 213 excluded records. Ultimately, 58 original studies on transcriptomics and proteomics related to KOA and HOA were included in this review. For details, see Table S2. The retrieval results are presented in the literature screening flow diagram (Figure 1).

### 3.2. Data Synthesis Rule

Based on the above retrieval strategies, we systematically extracted data from the 58 included OA omics original studies and compiled them into an evidence matrix (Table S2). Subsequently, the upregulated and downregulated differentially expressed factors in this matrix were ranked separately: factors with fold change (FC) values were sorted in descending order by |FC|, whereas factors without FC values were ordered according to the sequence of their narrative description in the original texts (as annotated in Table S2). Based on these rankings, the top 10 factors from each category were selected and presented in tables. respectively. Thereafter, to identify shared differentially expressed factors between knee and hip osteoarthritis, we used the complete evidence matrix (Table S2) as the basis, restricted the analysis to human tissues or cells, retained only factors that met the predefined significance threshold, removed duplicates, and excluded factors without a clearly reported direction of change. Finally, we performed a cross-comparison of differentially expressed factors between knee and hip to extract overlapping factors and summarize their expression trends (Table S3). Meanwhile, considering the heterogeneity in study design and platform among the included studies, we employed an adaptive quality assessment comprising six items, rather than a standardized risk-of-bias tool such as ROBINS-I (see Table S4 for details).

## 4. Common Differentially Expressed Factors (Co-DEFs) in Cartilage Samples from KOA and HOA Patients

According to the screening criteria described above, a total of five publications that performed comparative transcriptomic or proteomic analyses on cartilage samples from KOA and HOA were retrieved in this section. Among the literature using transcriptome sequencing, Xu et al. conducted the first comprehensive analysis at the transcriptomics level of human KOA and HOA cartilage, along with cartilage from patients with neck of femur fracture (NOF). The study found that KOA and HOA showed inconsistencies in individual gene expression but had commonalities in the involved molecular pathways. They summarized 81 DEGs that were simultaneously upregulated and 77 that were downregulated in both KOA and HOA [86]. Den Hollander et al. assessed gene expression differences in differentially methylated regions (DMRs), followed by transcriptomics analysis of DMR genes, revealing distinct expression patterns in the knee joint and hip joint. They identified 11 DEGs that were simultaneously upregulated in KOA and HOA, but the article did not mention simultaneously downregulated expressed genes [87]. By comparing the transcriptomics of lincRNAs in hip joint and knee joint cartilage, Ajekigbe et al. suggested that lincRNAs exhibit selective expression in cartilage. They screened out ten upregulated factors and one downregulated factor simultaneously in KOA and HOA cartilage, suggesting their involvement in cartilage homeostasis [88]. Hsueh and Casalone et al., on the other hand, performed a comparative analysis of the genome in KOA and HOA cartilage samples using proteomics sequencing technology. Hsueh et al. identified 177 differentially expressed proteins in KOA and HOA compared to normal cartilage tissue, but did not mention the expression trends of these differentially expressed proteins [89]. Different from the specimens tested by Hsueh et al., Casalone et al. performed proteomics analysis on chondrocytes from KOA and HOA, identifying 21 factors, but also did not mention the expression trends of these factors [90]. These co-DEFs are detailed in Table 1.

**Figure 2:**
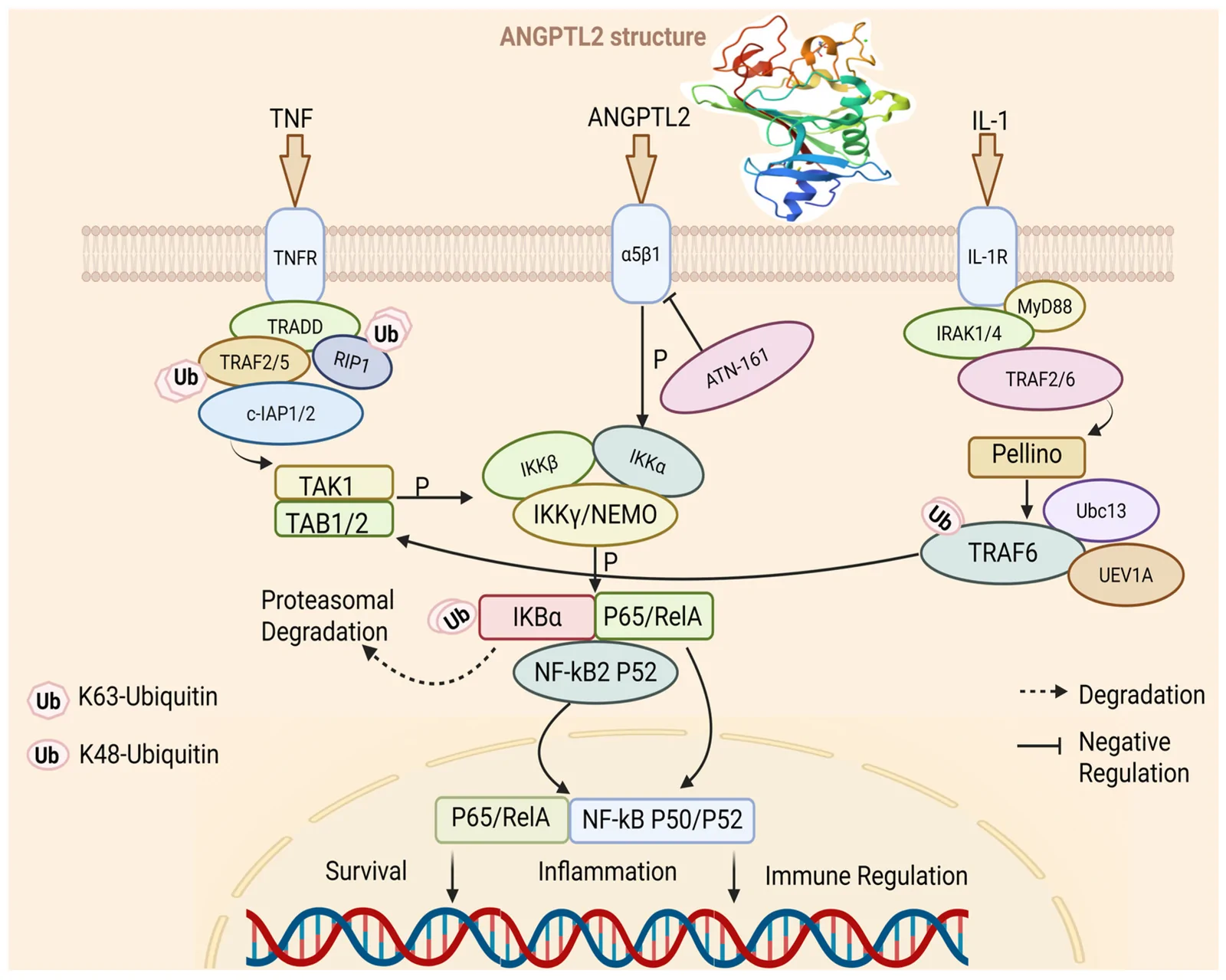
Schematic diagram of the related signaling pathways and structure of ANGPTL2 in osteoarthritis. The literature reports that TNF and IL-1 are two classic pathways in the NF-κB signaling pathway, which can activate the IKK complex. ANGPTL2 can also activate the IKK complex through integrin α5β1 and regulate the NF-κB signaling pathway. Furthermore, ATN-161, which inhibits integrin α5β1, significantly antagonizes the phosphorylation level of p65 in primary chondrocytes treated with ANGPTL2 overexpression. Created in BioRender. sun, Y. (2026) https://BioRender.com/10ac3jq.

According to Table 1, we conducted a detailed comparison of the factors mentioned in each article and found three factors that were frequently reported in the transcriptomics and proteome and could promote OA progression: ANGPTL2, CCDC80, and HTRA1. Angiopoietin-like 2 (ANGPTL2), a secreted glycoprotein, can accelerate tumor progression in some cancers [91,92]. Shan et al. first highlighted the negative role of ANGPTL2 secreted by human chondrocytes in osteoarthritis pathogenesis and its putative role as a therapeutic target for osteoarthritis (OA). They also found that ANGPTL2 may regulate the NF-κB signaling pathway through Integrin α5β1 and that ATN-161 can inhibit Integrin α5β1, significantly antagonizing the phosphorylation level of p65 in primary chondrocytes treated with ANGPTL2 overexpression [93]. The cell signaling pathway of ANGPTL2 is detailed in Figure 2. CCDC80, also known as coiled-coil domain containing 80 [94], has gene mutations that may be associated with stomach cancer [95], thyroid cancer [96], and inflammatory bowel disease [97]. Korthagen et al. discovered that CCDC80 may be a gene that marks the early stages of osteoarthritis progression in patients [98]. HTRA1, also known as serine protease 1, is expressed in the retina [99,100,101,102] and may be a target for age-related macular degeneration treatment [103]. Shi et al. found that knocking down HTRA1 can effectively inhibit myocardial fibrosis in dilated cardiomyopathy and improve left ventricular function [104]. Tsuchiya et al. found upregulation of HTRA1 in articular cartilage with osteoarthritis damage in mouse tissues [105], and Wilkinson et al. and Oka et al. found that HTRA1 inhibits TGFβ family protein-mediated signal transduction [106,107]. The mechanisms of these factors in osteoarthritis are not yet fully understood, and further in-depth exploration is expected to reveal new therapeutic targets for the disease, providing potential directions for future treatment strategies.

Due to the limited existing literature and the insufficient number of differentially expressed factors, it is still difficult to directly guide clinical practice. However, this research direction is of great significance for the development of diagnostic and treatment strategies for osteoarthritis. To this end, this study systematically retrieved and sorted the transcriptomics and proteomics literature in the PubMed database up to November 30, 2025 and further summarized the differentially expressed genes shared by KOA and HOA, aiming to provide a theoretical basis for the subsequent discovery of biomarkers and the establishment of therapeutic targets.

## 5. Multi-Omics Characteristic Molecules of KOA Cartilage

### 5.1. Differentially Expressed Genes (DEGs) in the Transcriptomics of KOA Cartilage

According to the screening criteria described above, a total of 23 original studies that conducted transcriptomic analyses on KOA cartilage samples were included in this section. Geyer et al. found that WISP-1, AQP-1, and DNER could be identified as novel target molecules and candidate biomarkers involved in the pathogenesis of osteoarthritis [108]. The findings of Rai et al. have supported molecular differences between medial and lateral compartments reflective of the greater severity of OA in the medial compartment [109]. The findings of Khan et al. demonstrated that GPR68 was robustly expressed in human cartilage and mice and its expression correlates with matrix degeneration and severity of OA progression in humans and surgical models [110]. Di et al. and Yuan et al. also used RNA sequencing to compare DEGs in the weight-bearing area versus the non-load-bearing area and the lateral region versus the medial region of KOA cartilage tissue, respectively. However, none of these three teams reported the expression trends of these DEGs [111,112]. Winstanley-Zarach et al. used RNA sequencing and identified multiple upregulated and downregulated DEGs associated with KOA [113]. Katsoula et al. identified 68 upregulated DEGs and 30 downregulated DEGs [114]. Han et al. employed RNA sequencing technology to comprehensively characterize the similarities and differences in mRNAs, lncRNAs, and circRNAs within the cartilage of Kashin–Beck disease (KBD) and osteoarthritis (OA) and identified certain non-coding RNAs as candidate diagnostic biomarkers or therapeutic targets for KBD patients [115]. This study by Mei et al. further advances the understanding of the interplay between genes, chemicals, and their impact on osteoarthritis [116]. The Gene Expression Omnibus (GEO), a public gene expression database created and maintained by the National Center for Biotechnology Information (NCBI), has collected high-throughput genomic data submitted by research institutions worldwide since 2000, encompassing data generated by technologies such as microarray chips, next-generation sequencing, and single-cell sequencing [117,118,119,120]. Li et al. performed differential analysis and functional enrichment analysis using KOA cartilage-related datasets from GEO, initially comparing female KOA with female healthy knee cartilage tissue and male KOA with male healthy knee cartilage tissue, identifying multiple KOA-related DEGs [121,122]. Tang et al. also utilized GEO, discovering 10 upregulated and 10 downregulated DEGs in the cartilage tissue of KOA patients [123].

Cartilage tissue is composed of chondrocytes, a matrix, and fibers [124,125,126]. Unlike the sample sources in the aforementioned studies, other studies have used RNA sequencing or single-cell RNA sequencing techniques to identify relevant DEGs in chondrocytes from patients with KOA. Chen et al. suggested that novel miRNA regulation plays a key role in the pathogenesis of knee joint microenvironment alterations in osteoarthritis (OA) [127]. Steinberg et al. and Wu et al. used RNA sequencing to analyze degenerate cartilage and intact cartilage from KOA and severely damaged cartilage and intact smooth cartilage from KOA, respectively, discovering several upregulated and downregulated DEGs [128,129]. Single-cell RNA sequencing, a high-throughput sequencing technology in biology, is used to analyze gene sequences, structures, and expression at the single-cell level to reveal differences in genetic information between cells. The first human single-cell sequencing study was completed in 2012. Tang et al. were the first to utilize single-cell RNA sequencing (scRNA-seq) to elucidate the transcriptomic alterations and distinct cell subtype populations in the cartilage of subchondral insufficiency fracture of the knee (SIFK) and osteoarthritis (OA), providing new perspectives on the pathogenesis and progression of SIFK and OA [130]. Fan et al. employed single-cell RNA sequencing to analyze the heterogeneity of knee chondrocytes and identified potential cell populations for targeted therapy [131]. Sun et al. used single-cell RNA sequencing technology to verify the marker genes of seven chondrocyte subtypes [132].

Some scholars have also investigated cartilage tissue or cells from mouse or rat KOA models to identify key genes associated with KOA cartilage. Sebastian et al. identified a total of 29 upregulated and 39 downregulated DEGs associated with cartilage tissue in KOA [133]. Sebastian et al. performed OA modeling on C57BL/6J mice and, for the first time, used single-cell RNA sequencing technology, discovering five upregulated and four downregulated DEGs [134]. Wu et al., Zhao et al., and Hu et al. performed OA modeling on SD rats, Wistar rats, and Lewis rats, respectively, and used RNA sequencing technology to analyze DEGs related to KOA [135,136,137]. These DEGs are detailed in Table 2.

### 5.2. Differentially Expressed Proteins (DEPs) in the Proteomics of KOA Cartilage

According to the screening criteria described above, a total of 19 original studies that conducted proteomic analyses on KOA cartilage samples were included in this section. Hermansson et al. used two-dimensional gel electrophoresis–mass spectrometry (2-DE/MS) technology to analyze 11DEPs in KOA cartilage tissue, but they did not report the expression trends of these DEPs [138]. Guo et al. also used the same technology and found 14 upregulated DEPs in KOA cartilage tissue [139]. Di et al. compared cartilage tissue from the weight-bearing region and non-weight-bearing region of KOA and found 28 upregulated proteins and 29 downregulated proteins, providing new ideas for promoting the repair of cartilage damage [111]. 2-DE is particularly suitable for rapid comparison of protein expression differences between two samples, but it also has shortcomings such as being time-consuming and labor-intensive, with limited sensitivity. Liquid chromatography–tandem mass spectrometry (LC-MS/MS) compensates for these shortcomings, as it has advantages such as high throughput, high sensitivity, and automation. Currently, many proteomics studies have shifted to LC-MS/MS. Garcia et al. first used LC-MS/MS technology and discovered related proteins in KOA cartilage tissue. However, this study did not mention a healthy control sample [140]. Chen et al. performed a proteomics analysis of 15 KOA and 11 healthy knee joint articular cartilage tissues, and they found 364 significantly upregulated and 424 downregulated DEPs [141]. Steijns et al. treated KOA patients with knee joint distraction (KJD) and analyzed their cartilage tissue after the procedure, identifying 25 upregulated and 21 downregulated DEPs [142]. Fan et al. summarized the top ten hub proteins in the protein–protein interaction (PPI) network of KOA cartilage tissue and found that changes in calcified cartilage could reflect the progression of KOA [143]. Brumwell et al. identified proteomic changes in the phenotype of human primary articular chondrocytes (hPACs) from OA patients after collagenase digestion. They found a total of 375 upregulated DEPs and 123 downregulated DEPs. Ultimately, they advocated for tissue fixation prior to nucleic acid extraction for analysis [144].

**Table 2:** Differentially expressed genes in the KOA transcriptome (top 10 DEGs listed).

Sample Type	Species	Experimental Group	Omics Platform and Technology	Statistical Methods	Top 10 DEGs		Year	References
					Upregulated	Downregulated		
Cartilage Tissue	Human	OA (n = 5) vs. NC (n = 5) Male = 1 Female = 4	Affymetrix HG-U133 Plus 2.0 array	Array differential expression (SLR)	AQP-1, DAF, DNER, IF, IGFBP-3, WISP-1 Sort by: Alphabetical	Not mentioned	2008	[108]
Cartilage Tissue	Human	OA medial compartment cartilage (n = 23) vs. OA lateral compartment cartilage (n = 23) Male = 10 Female = 13	Affymetrix Gene 2.0 ST Array	*t*-test for *p* values and fold changes	MMP13, MATN3, EPYC, SCARNA5, COL2A1, SNORA11, SCARNA14, HISTIH2BM, LECT1, COLIIA1	SELE, MCTP1, CTSS, VSIG4, F13A1, STEAP4, SLCO2A1, GUCYIA3, TEK, IFI27	2018	[109]
Cartilage Tissue	Human	OA (n = 20) vs. NC(n = 18)	limma differential expression (logCPM)	One-way ANOVA; Tukey; paired *t*-test	ADAMTS4, ADAMTS5, GPR68, MMP3, MMP9, MMP13	Not mentioned	2023	[110]
Cartilage Tissue	Human	OA weight-bearing area (n = 28) vs. OA non-load-bearing area (n = 28)	Illumina HiSeq RNA-seq	Wilcoxon rank-sum test (scRNA-seq)	SLC39A14, FTH1	TGFB1	2023	[111]
Cartilage Tissue	Human	Lateral OA vs. medial OA	Illumina RNA-seq	Student’s *t*-test; One-way ANOVA	SULF1, HS6ST3, SDC2, HS2ST1, HS6ST2, HS6ST1, HS3ST1, CHST13, EXT2, HSPG2 (trends not mentioned)		2025	[112]
Cartilage Tissue	Human	Male OA (n = 6) vs. Male NC (n = 6)	QuantSeq-Reverse 3′ RNA-seq	One-way ANOVA	AC008592.4, PCSK1, AL355596.1, AC083843.3, CYP4B1, LINC01554, RIPOR2, GASAL1, MRC1, NOD2	GRIN2A, ADAMTS2, AL139220.2, MGAT3, CLEC3A, TNFRSF18, GREM1, MATN3, FAM180B, GRIA2,	2023	[113]
Cartilage Tissue	Human	High-grade OA cartilage (n = 124) vs. low-grade OA cartilage (n = 124)	Illumina TruSeq RNA	Benjamini–Hochberg correction	OGN, RN7SL376P, R3HDML, HTRA1, CRLF1, TMEM59L, JSM2, FSTL3, EVI2A, EVI2B	SOX7, CFH, FRZB, CHRDL2, RCAN2, SPOCK3, ViT, GDF7, WNK2, MATN4	2022	[114]
Cartilage Tissue	Human	OA (n = 10, Male = 2 Female = 6) vs. KBD (n = 8, Male = 3 Female = 7)	Illumina Hiseq 4000 (Illumina, Inc., San Diego, California, USA.)	Paired *t*-test	KRT6A, SFT203, AKAJN1, KRT16, DR2T2, KCNJ16, MSMP, DUX4, SAA1	KLK1, EMFD3, PTH1R	2024	[115]
Cartilage Tissue	Human	OA (n = 20) vs. NC (n = 18)	TWAS and CGSEA	TWAS gene association	EVI2A, LTBP1, CDK2AP1, GDF5	WWP2, SPAG4	2023	[116]
Cartilage Tissue	Human	Female OA (n = 11) vs. Female NC (n = 5) Male OA (n = 9) vs. Male NC (n = 13)	limma-voom differential expression	Quasi-likelihood F-tests	POSTN, TNFAIP6, THY1, COL1A1, ST6GALNAC5, TNFSF15, NGF, MKI67, NOTCH3, COL1A2 ST6GALNAC5, TNFSF15, CFI, SPOCK1	DDIT4, KIT, ADM, SIK1B, MAFF, MYO7A, FAM166A, ATF3, HILPDA, ALDH1L1 ADM, HILPDA, ATF3, ANGPTL4, DDIT4, SIK1B, CISH, JUN, LOC105371934, GADD45B	2021	[121]
Cartilage Tissue	Human	Female OA (n = 11) vs. Female NC (n = 5) Male OA (n = 9) vs. Male NC (n = 13)	limma-voom differential expression	Quasi-likelihood F-tests	TNFAIP6, THY1, COL1A1, ST6GALNAC5, TNFSF15, COL1A2, MXRA5, TNC, COL3A1, DIO2 POSTN, ST6GALNAC5, TNFSF15, LRRC15, COL1A1, GRIA2, TREM1, CDH2, ASPM, LOC100129940	DDIT4, KIT, ADM, SMAFF, MYO7A, FAM166A, HILPDA, TXNIP, DDIT3, CISH ADM, HILPDA, ATF3, ANGPTL4, DDIT4, CISH, JUN, LOC105371934, GADD45B, BHLHE40	2021	[122]
Cartilage Tissue	Human	OA (n = 20) vs. NC (n = 18)	DESeq2 differential expression	Spearman correlation	POSTN, CSN1S1, COL1A1, IGFBP1, HBB, IL11, VSIG4, HCN1, C1QA, RAC2	RND1, CYP4B1, DUSP2, ADM, HILPDA, MIR5087, HIST1H1D, ELF3, ATF3, DDIT4	2024	[123]
Chondrocyte	Human	OA vs. NC	Illumina Solexa 75 bp single-end	Mann–Whitney U test	COL18A1, APOE, RGS4, ERAP2, B4GALNT4, ALPL, FGF5, LMOD1, RGS5, OLFM2		2018	[127]
Chondrocyte	Human	OA degenerated cartilage vs. OA intact cartilage	Illumina HiSeq 2000 (Illumina, Inc., San Diego, California, USA.); Illumina 450K BeadChip	paired *t*-test	PODN, R3HDML, POSTN, CHRDL1, NPR3, SFRP2, TFEC, COL1A1, CCR1, CCR7	DEFA1B, LTF, UCMA, RHAG, CHRDL2, MATN4, PMCH, FAM90A1, DEFB104B, PLCXD3	2017	[128]
Chondrocyte	Human	OA severely damaged cartilage (G4) (n = 20) vs. OA intact smooth cartilage (G1) (n = 20) Male = 8 Female = 12	RNA sequencing	Paired *t*-test, two-way ANOVA + Tukey post hoc	IL36RN, R3HDML, WNT16, AREG, CYP4F29P, PKHD1, TREML4, AC034223.2, AC018685.2, APLNR	MLC1, HAO1, KLK10, LNCNEF, CHAD, MAFAIL2RG, LRRC38, TAGLN3, TMEM132C, CCL20	2022	[129]
Chondrocyte	Human	Female OA (n = 11) vs. Female SIFK (n = 11)	Single-cell RNA sequencing; Illumina HiSeq (Illumina, Inc., San Diego, California, USA.)	Differential proportion tests	SERPINE2, TNFRSF11B, MMP3, CCL20, G0S2, LCN2, CXCL8, S100A2, TNFAIP6, HBB	COL1A2, PTN, COL1A1, SPARC, COL11A1, CRISPLD1, COL14A1, COL9A1, COL2A1, IBSP	2022	[130]
PreHTC Chondrocytes	Human	OA (n = 8) vs. NC (n = 3)	Single-cell RNA sequencing; Illumina NovaSeq 6000 (Illumina, Inc., San Diego, California, USA.)	Benjamini–Hochberg (FDR)	POSTN, THY1, AQP1, COL1A2, THBS2, CELF2, COL3A1, CRLF1, GFRA2, ASPN	SAA1, LCN2, MMP3, SLPI, G0S2, STC2, APOD, CCDC71L, DDIT4, STEAP4	2023	[131]
Chondrocyte	Human	OA (n = 3) vs. NC (n = 3)	10x Genomics Chromium Single-Cell 3′ v3 + Illumina sequencing	Seurat FindMarkers (Wilcoxon rank-sum test); *t*-test (subtype proportions)	7 subtype marker genes: EC-A: CHRDL2, EC-B: COL2A1, HTC-A: HILPDA, HTC-B: IBSP, RegC-B: VCAM1, METRNL+: METRNL, ANGPTL4, PRG4+: PRG4, CRLF1		2024	[132]
Cartilage Tissue	Mice	Aged male mice with non-invasive tibial compression injury vs. Young male mice	Illumina TruSeq RNA + NextSeq 500	Seurat analysis (scRNA-seq); paired *t*-test	Ccl3, II10, II17re, H2-Eb1, Cxcl9, 115ra, Cd6, Cd51, Cxcr6, Cxcr3	Bmp8a, Pth1r, Runx2, Fgfr3, Comp, Gli2, Col1a1, Chadl, Matn1, Satb2	2020	[133]
Chondrocyte	C57BL/6J Mice	Male OA mice (n = 5) vs. Male NC mice (n = 5)	Illumina TruSeq RNA + NextSeq 500	RUVseq; Seurat analysis (scRNA-seq); paired *t*-test	Mmp3, Mmp13, Slpi, Col10a1	Col9a1-a3, Sox9, Prelp, Il17b, Cilp	2021	[134]
Cartilage Tissue	SD Rats	Mechanical load male OA rats (n = 3) vs. NC (n = 3)	Illumina RNA-seq	ANOVA or two-way ANOVA + Tukey post hoc	IL-6, IL-β, NLRP3, iNOS, TNF-α, MMP13, ADAMTS4	IL-10, TGF-β, ACAN, COL2A1	2025	[135]
Cartilage Tissue	Wistar Rats	6-month-old male OA rats (n = 8) vs. 12-week male OA rats (n = 8)	Illumina RNA-seq	Mann–Whitney U test	Kcnj4, Csmd2, Kcnk3, Amigo2, Grtp1, Serpina12	Lilrb3a, Anpep, cd44, Igfbp3, Ctsk, Acp5, C7, Mcpt10, Cyfip2, Tf	2023	[136]
Cartilage Tissue	Lewis Rats	6-week post-MMT male OA rats vs. NC	Illumina RNA-seq	One-way ANOVA; Kruskal–Wallis test	Ngf, Crlf1, Tnn, Krt13, Serinc2, Clec2dl1, Inhba, Serpine1, Aldh1a3, Col3a1	Cpz, Nnat, Lmo2, Ccdc88c, Irf7, Notum, Myoc, Dlk1, Cxcl13	2022	[137]

KBD, Kashin–Beck disease; TWAS and CGSEA, transcriptome-wide association study and cluster-based gene set enrichment analysis; GEO, Gene Expression Omnibus; SIFK, subchondral incomplete fracture of the knee joint; preHTC, prehypertrophic chondrocyte; EC-A, effector chondrocytes type A; EC-B, effector chondrocytes type B; HTC-A, homeostatic chondrocytes type A; HTC-B, homeostatic chondrocytes type B; RegC-B, regulatory chondrocytes type B; METRNL+, METRNL-positive chondrocytes; PRG4+, PRG4-positive chondrocytes; MMT, medial meniscus transection.

Unlike the aforementioned studies that used different sample sources, other studies have sourced human chondrocytes. Lambrecht et al. revealed major alterations in the vimentin cytoskeleton in human articular chondrocytes affected by OA [145]. Yu et al. compared H2O2-treated OA chondrocytes with HA/H2O2-treated OA chondrocytes, using 2-DE/DM technology to identify 13 KOA-related DEPs, but did not mention their expression trends [146]. Tsolis et al. compared quantitative changes in protein synthesis between osteoarthritic chondrocytes and normal chondrocytes. They identified several pathways and proteins associated with osteoarthritic chondrocytes, providing evidence for further exploration of the molecular mechanisms underlying this disease [147]. Steinberg et al. also used LC-MS/MS to compare degenerate cartilage with intact cartilage in KOA, identifying numerous KOA-related DEPs, but they did not mention expression trends [128]. Wu et al. compared severely damaged cartilage (G4) with intact smooth cartilage (G1) in KOA, identifying 73 upregulated DEGs and 57 downregulated DEGs [129]. Balaskas et al. used LC-MS/MS proteomics to summarize a large number of KOA DEPs, but they did not specify expression trends [148]. Hsueh et al. identified candidate biomarkers for osteoarthritis (OA) and its progression, as well as joint-specific biomarkers [89]. Destouni et al. identified 24 upregulated and 81 downregulated differentially expressed proteins in KOA chondrocytes using one-dimensional gel electrophoresis-nano reversed-phase liquid chromatography (1-DE/RP-LC) techniques [149]. Chen et al. applied proteomics techniques to cartilage samples from damaged areas and intact cartilage of knee joints in osteoarthritis patients, discovering DEPs in KOA and clarifying their expression trends [150].

In addition, Li et al. performed proteomics analysis on knee cartilage tissue from an anterior cruciate ligament reconstruction (ACLR) mini-pig model, identifying a total of 491 DEPs, including 198 that were upregulated and 293 that were downregulated. Although the original text did not list specific gene names, it summarized potential genes related to KOA [151]. Fongsodsri et al. used LC-MS/MS technology with mouse chondrocytes and summarized several downregulated DEPs associated with KOA [152]. These DEPs are detailed in Table 3.

## 6. Multi-Omics Characteristic Molecules of HOA Cartilage

### 6.1. Differentially Expressed Genes (DEGs) in the Transcriptomics of HOA Cartilage

According to the screening criteria described above, a total of eight original studies that conducted transcriptomic analyses on HOA cartilage samples are included in this section. Femoroacetabular impingement (FAI) is considered a pre-OA condition, playing an etiological role in up to 50% of HOA cases [153,154,155]. Pascual-Garrido et al. discovered key molecules in the progression of advanced HOA and early FAI patients and identified 31 upregulated and 17 downregulated DEGs associated with HOA [156]. Research by Kuhns et al. has further expanded the understanding that distinct genetic reprogramming mechanisms exist in the cartilage of patients with FAI and HOA [157]. Huang et al. found that HOA can be classified into two distinct molecular subtypes. The main differences between these two subtypes lie in changes in pro-inflammatory factors and energy metabolism [158]. Huang et al. performed RNA-seq analysis on a large number of samples and identified multiple DEGs [159]. Steinberg et al. discovered potential molecular changes during the progression of HOA and identified 86 upregulated and 18 downregulated DEGs [128]. Steinberg et al. used RNA sequencing to identify multiple DEGs in osteoarthritic chondrocytes and low-grade chondrocytes from HOA patients, providing new insights into the biological processes of osteophyte development and osteoarthritis progression [160]. Aki et al. first reported the whole-genome transcriptome of chondrocytes in secondary HOA and identified 22 upregulated and 12 downregulated DEGs in secondary HOA compared to healthy chondrocytes using microarray analysis [161]. Southan et al. performed microarray analysis on mouse hip joint epiphyseal cartilage tissue before and after injury, revealing relevant DEGs and significant regulation of metabolic responses following mechanical injury to the articular cartilage [162]. The above DEGs are detailed in Table 4.

### 6.2. Differentially Expressed Proteins (DEPs) in the Proteomics of HOA Cartilage

According to the screening criteria described above, a total of four original studies that conducted proteomic analyses on HOA cartilage samples were included in this section. In the field of osteoarthritis research, the hip joint receives less academic attention and research investment compared to the knee joint. It is hoped that more people will conduct more in-depth explorations of HOA in the future. Steinberg et al. used liquid chromatography–mass spectrometry (LC-MS) analysis technology to extract a large number of DEPs related to HOA and clarified their expression trends [128,160]. Hosseininia et al. identified seven DEGs that were upregulated and suggested that these genes may represent early molecular changes during the progression of HOA, with the potential to predict the development of HOA [163]. Hsueh et al. analyzed HOA cartilage tissue using reversed-phase liquid chromatography (RPLC) and identified significantly enriched proteins in HOA cartilage. However, they did not mention their expression trends [164]. The above DEPs are detailed in Table 5.

**Table 4:** Differentially expressed genes in the HOA transcriptome (top 10 DEGs listed).

Sample Type	Species	Experimental Group	Omics Platforms	Statistical Methods	Top 10 DEGs		Year	References
					Upregulated	Downregulated		
Cartilage Tissue	Human	FAI-OA late (n = 13, Male = 9 Female = 4) vs. FAI-OA early (n = 9, Male = 6 Female = 3)	Illumina NovaSeq 6000 RNA-seq	Mann–Whitney U; ANOVA; Kruskal–Wallis	TREMI, GUCAIA, PTGES, LINCO1614, HILPDA, NGPTL4, VEGFA, SERPINE1, TNFAIP6, IER3	TCA1, TPRG1, MYOC, LMO3, HIF3α, PDZD8, B3GALT1, SORL1, SCN4A, ARGLU1	2022	[156]
Cartilage Tissue	Human	OA (n = 10) vs. FAI (n = 10) Male = 5 Female = 5	RNA sequencing	Paired *t*-test	TNF, FGF23, MATN4, IL1R1, ADAMTS4, MMP11, COL10A1, COL11A1, COL9A1, FRZB	IL17D, BMPR1B, FGF18, WNT16	2023	[157]
Cartilage Tissue	Human	OA (n = 100, Male = 50 Female = 50) vs. NC (n = 120, Male = 49 Female = 71)	Illumina Omni-2.5 genotyping	Wilcoxon; Student’s *t*-test; ANOVA	C12orf75, TMEM117, ADCY1, NUAK1, SH3D19, TENM3, STXBP6, DCLK2, ZNF503, METTL2B	MARCHF3, TGIF1, HILPDA, ZNF385D	2024	[158]
Cartilage Tissue	Human	OA (n = 72, Male = 32 Female = 40) vs. NC (n = 12, Male = 3 Female = 9)	Illumina NovaSeq 6000 RNA-seq	Wilcoxon	MARCHF3, PNRC1, S100A4, KDM7A, ELL2, GCH1, RPGR, SLC25A28, NAMPT, SPARC (trends not mentioned)		2025	[159]
Chondrocyte	Human	Degenerated cartilage OA vs. intact cartilage OA	Illumina HiSeq 2000; Illumina 450K BeadChip	Paired *t*-test	KRT17, NPR3, R3HDML, TMEM59L, TREM1, NOTCH3, MEOX2, CCBE1, ORM1, DNAJC12	SPOCK3, FAM78B, PMCH, PLCXD3, CHRDL2, HAO1, S100A16, FABP5P7, CDHR1, PTGER3	2017	[128]
Chondrocyte	Human	Osteophytic OA (n = 9) vs. low-grade OA (n = 9) Male = 3 Female = 3	Illumina HiSeq 2000	MAGMA; one-sided hypergeometric test	HAO1, SPTSSB, SFN, KIF1A, FST, CNTFR, PLCXD3, AC129778.2, RP11-381O7.3, HS3ST3A1	ITGAX, ALX4, HCK, LBP, STAT4, SDK1, RAC2, MEOX2, KCNK2, HAS1	2018	[160]
Chondrocyte	Human	OA (n = 12, Male = 1 Female = 11) vs. NC (n = 8, Male = 1 Female = 7)	Illumina Human HT-12 V3 array	Mann–Whitney U test	COL1A1, ASPN, TPPP3, OGN, COL3A1, MXRA5, DPT, AQP1, CRIP1, TGFB1	PDZK1, GPX3, BEX2, HIST1H1C, STEAP4, CP, CCL20, C10OrF10, C4BPA, LCN2	2018	[161]
Cartilage Tissue	Mice	Degenerative OA vs. NC	Illumina mouse BeadChip array	Student’s *t*-test	Has2, Arg1, Eno1, Upp1, Slc2a1, Sc7a1	Slc9a9, Slc37a1, Dcn, Eno3, Pgam2	2020	[162]

FAI: Femoroacetabular impingement.

**Table 5:** Differentially expressed proteins in the HOA proteome (top 10 DEPs listed).

Sample Type	Species	Experimental Group	Omics Platforms	Statistical Methods	Top 10 DEPs		Year	References
					Upregulated	Downregulated		
Cartilage Tissue	Human	OA with degenerated cartilage versus vs. OA with intact cartilage	LC-MS/MS	Paired *t*-test	AQP1, CLEC3B, PDLIM1, CLIC3, MAP1A	CHRDL2, TINAGL1, FGFR2, TBX4	2017	[128]
Cartilage Tissue	Human	Osteophytic OA (n = 9) vs. Low-grade OA (n = 9) Male = 3 Female = 6	LC-MS	MAGMA; one-sided hypergeometric test	VAT1L, MMP1, OXCT1, CFB, COX4I1, NDUFB7, NDUFS5	GDF10, GPX3, SCRG1, HTRA3, ALDH1A2, ADSSL1, HSPG2, CILP2, ACAN, ITIH6	2018	[160]
Cartilage Tissue	Human	OA (n = 9, Male = 4 Female = 5) vs. NC (n = 12, Male = 5 Female = 7)	LC-MS TSQ Vantage	Mann–Whitney U test	ASPN, MIME, MATN3, CILP-2, Collagen VI, Collagen II, and Collagen III N-terminal precursor proteins	Not mentioned	2019	[163]
Cartilage Tissue	Human	OA vs. NC	RPLC-MS	Multivariate regression; Holm step-down	CHAD, MGP, COBA2, TSP4, COL3-neo	COMP, CILP1-2, HTRA1, PGCA-1/2, PGS2, TARSH, TSP1	2018	[164]

LC-MS, liquid chromatography–mass spectrometry; RPLC (reverse-phase LC)-MS, reversed-phase liquid chromatography–mass spectrometry.

## 7. Screening and Characterization of Candidate Molecules for Osteoarthritis

**Figure 3:**
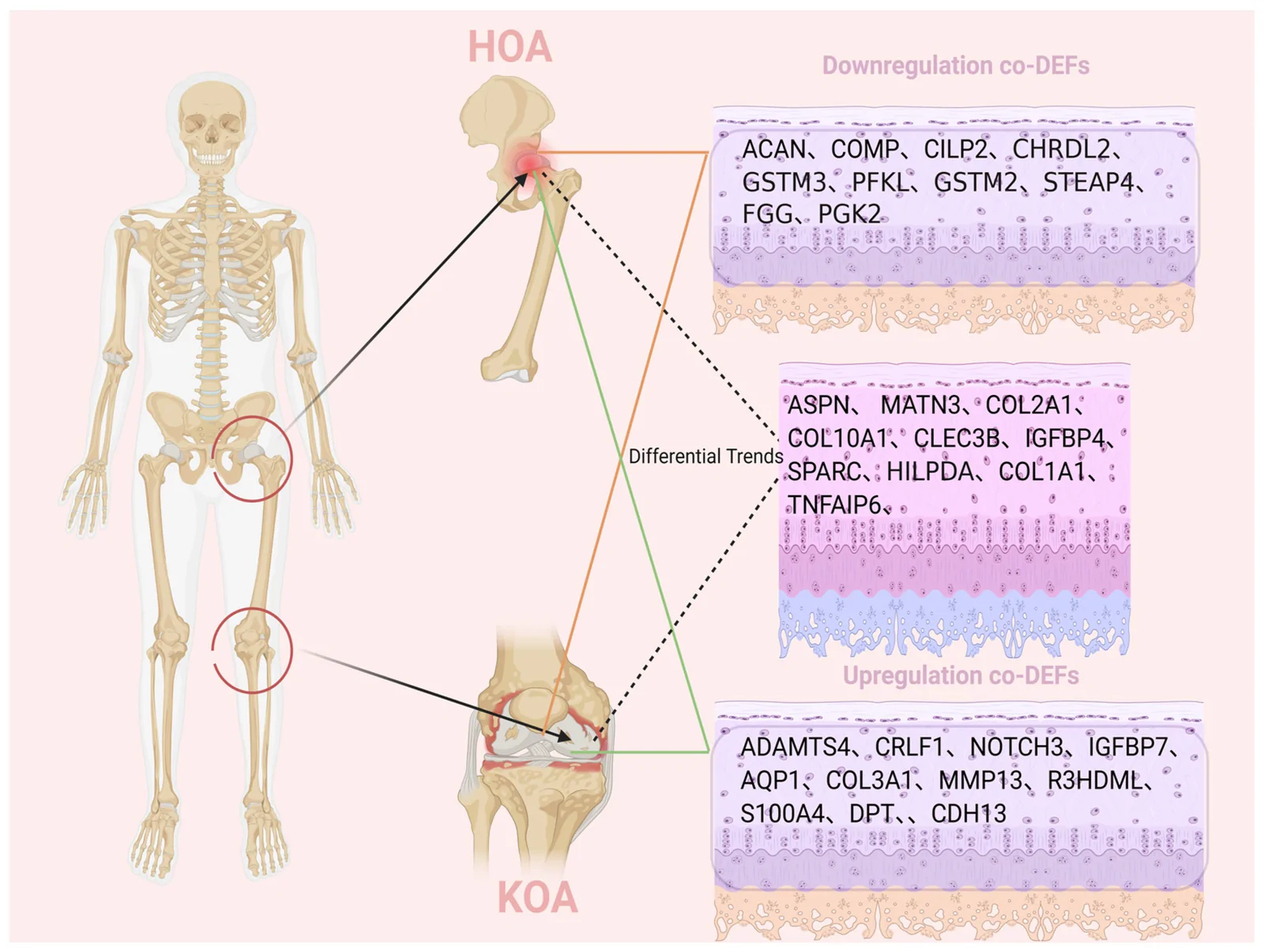
Expression trends of 31 common differentially expressed factors (co-DEFs) in KOA and HOA Compared to the control group, KOA and HOA shared 21 co-DEFs with identical expression trends. Among these, 10 factors, including ACAN and COMP, were downregulated in OA, whereas 11 factors, including ADAMTS4 and CRLF1, were upregulated in OA. In addition, 10 factors, including ASPN and MATN3, exhibited divergent expression trends between KOA and HOA, suggesting that these factors may be subject to differential regulatory mechanisms in distinct joints. This finding implies the potential for developing joint-specific therapeutic strategies in the future. Created in BioRender. sun, Y. (2026) https://BioRender.com/wer4hjw.

This article further summarizes the common differentially expressed genes in KOA and HOA, and a total of 31 common differentially expressed factors (co-DEFs) of KOA and HOA were screened. Among them, 10 factors showed a decreasing trend in both KOA and HOA, 11 factors showed an increasing trend in both KOA and HOA, and the trends of the remaining 10 factors are inconsistent between KOA and HOA. The above co-DEFs are detailed in Figure 3.

**Figure 4:**
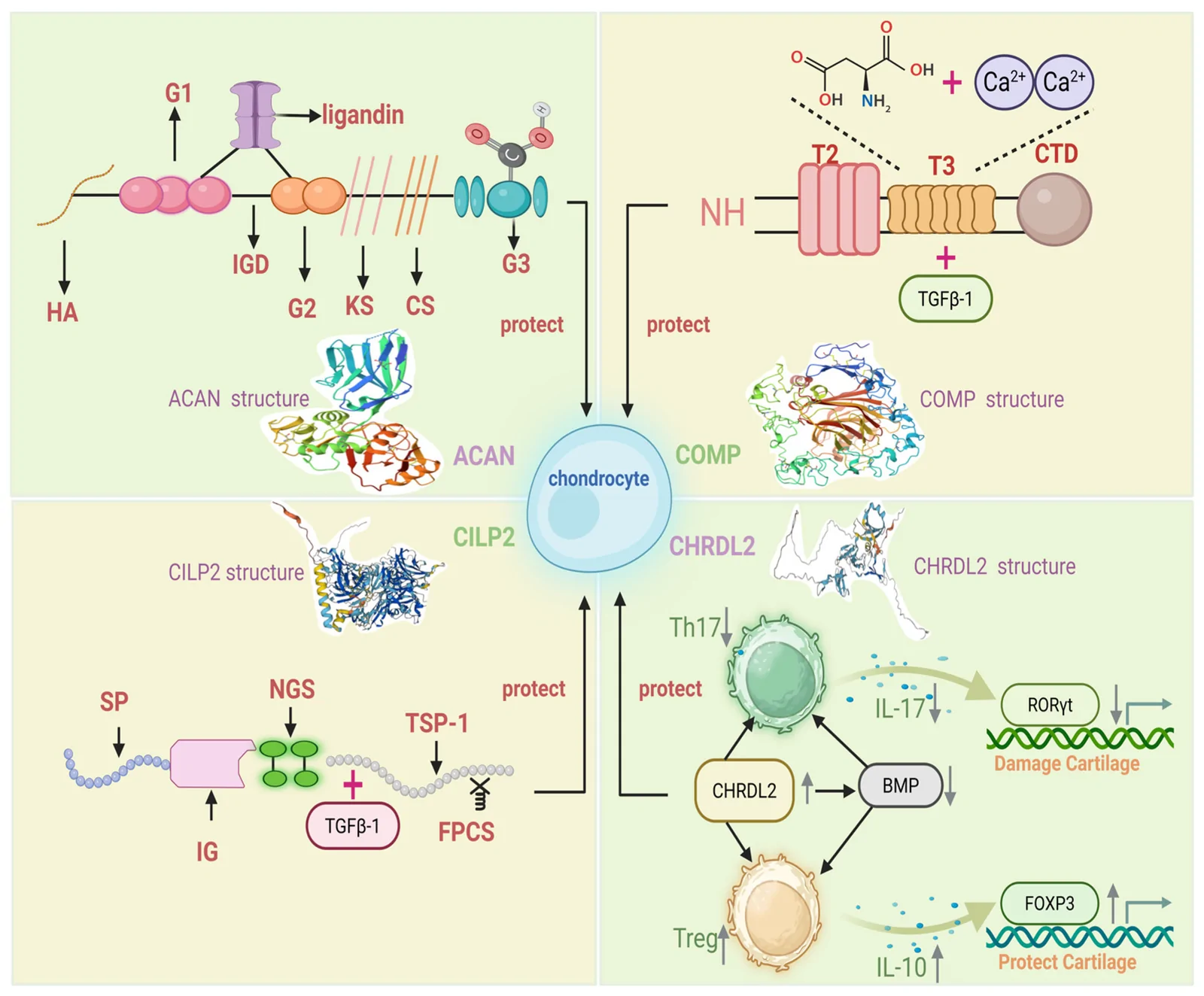
Structures of representative co-DEFs downregulated in KOA and HOA, and a schematic diagram illustrating the alleviation of OA and the protection of cartilage. Created in BioRender. sun, Y. (2026) https://BioRender.com/mgfdx1k.

The 10 co-DEFs that are downregulated in OA may serve as putative therapeutic targets for OA following subsequent validation experiments. Among them, ACAN and COMP are the most extensively studied factors that are critical for cartilage function, while limited studies suggest associations between CILP2, CHRDL2, and osteoarthritis. Currently, no experimental studies in the OA field exist for the remaining factors. ACAN, which stands for aggrecan, is a member of the multifunctional proteoglycan family and serves as the principal proteoglycan in articular cartilage [165]. The ACAN core protein consists of three globular domains (G1, G2, and G3) and three extended domains (IGD, KS, and CS). IGD connects G1 and G2. Located between G2 and G3 is a large sequence modified by KS and CS side chains. G1 interacts with hyaluronic acid (HA), G1 and G2 interact with link proteins, and G3 constitutes the carboxyl terminus of the core protein. Post-translationally, this protein is extensively modified by the addition of glycosaminoglycan (GAG) chains [166], primarily chondroitin sulfate and keratan sulfate. It provides a hydrated gel structure that confers load-bearing properties to cartilage, enabling it to withstand compression [167,168,169]. ACAN is also a key structural protein in the cartilage extracellular matrix, and its degradation may contribute to the development of OA [170]. A series of factors for assessing ACAN degradation have been identified [171], and multiple studies have shown that inhibiting this degradation to elevate ACAN levels can significantly improve patients’ mobility and functional status [172,173,174]. COMP, which stands for cartilage oligomeric matrix protein, encodes a non-collagenous extracellular matrix protein that is primarily present in the human skeletal system [175,176,177]. Each monomeric arm of COMP contains an N-terminal domain, four type 2 (T2) epidermal growth factor-like repeat sequences, seven type 3 (T3) repeat sequences, and a C-terminal globular domain (CTD). The T2 and T3 repeat sequences and CTD bind calcium ions. The T3 repeat sequences typically contain five aspartic acid residues per motif, with each motif enveloping two calcium ions. The binding of TGF-β1 to COMP enhances the TGF-β1-induced signaling pathway within chondrocytes. COMP can attract chondrocytes and may contribute to the regeneration of the damaged cartilage area. Maly et al. found that COMP can attract chondrocytes to promote repopulation of damaged cartilage areas and it also activates mechanisms that protect and repair the articular cartilage extracellular matrix [178]. Istiak et al. found that COMP indirectly protects cartilage from all applied proteases, thereby helping to preserve the mechanical integrity of cartilage [179]. CILP2, which stands for cartilage intermediate layer protein 2, is located in extracellular vesicles. As a secretory glycoprotein, CILP2 contains a signal peptide at the N-terminus, a core region including an immunoglobulin-like domain, a TSP-1 repeat sequence domain, which mediates the binding of TGF-β1 and matrix proteins, and distributed cysteine residues, a predicted N-glycosylation site, and a furin protease cleavage site; each domain synergistically participates in its function in the cartilage matrix. Bernardo et al. found that CILP2 is expressed in articular and meniscus cartilage and is downregulated in osteoarthritis [180]. CHRDL2, which stands for chordin-like 2, is a secreted protein. Li et al. found that overexpression of CHRDL2 corrects the Th17/Treg imbalance, reduces IL-17 and RORγt levels, and increases IL-10 and Foxp3 levels. CHRDL2 overexpression reduces BMP signaling activity, inhibits Th17 polarization, and promotes Treg development, thereby inhibiting OA progression and protecting cartilage [181]. Research on the remaining factors in the OA field remains insufficient and requires further in-depth exploration in the future. A schematic diagram illustrating the structures of these four factors as well as their roles in alleviating OA and protecting cartilage is shown in Figure 4.

**Figure 5:**
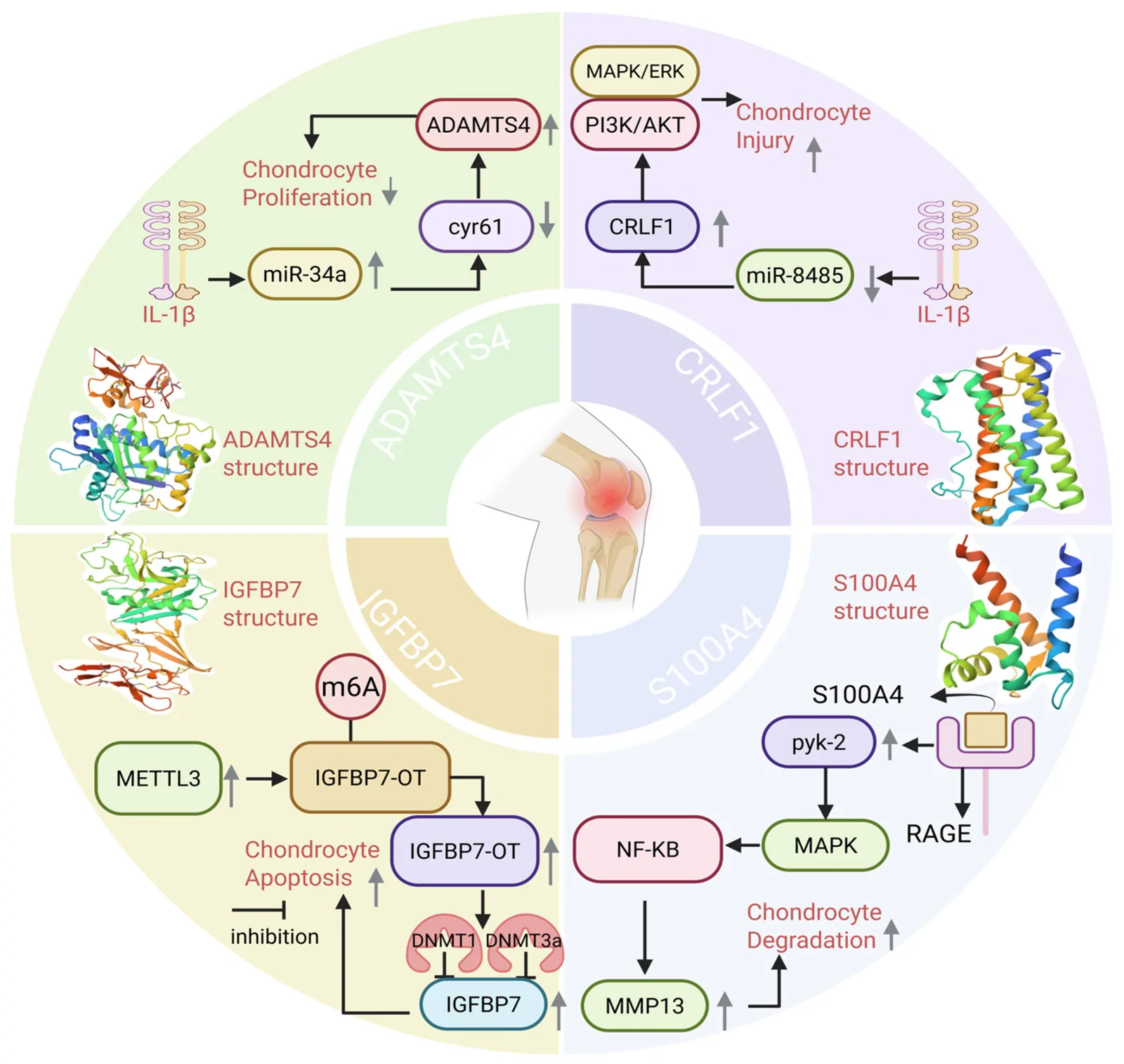
Structure of representative co-DEFs upregulated in KOA and HOA, and a schematic diagram illustrating the promotion of OA and the destruction of cartilage. ADAMTS4: Schematic diagram of ADAMTS4 structure and damaged cartilage. The figure reveals the critical role of the miR-34a/Cyr61/ADAMTS4 signaling axis in the pathogenesis of OA. IL-1β promotes miR-34a expression, miR-34a targets and inhibits Cyr61, Cyr61 negatively regulates ADAMTS4, and ADAMTS4 inhibits chondrocyte proliferation. IL-1β affects OA progression through the miR-34a/Cyr61/ADAMTS4 axis. CRLF1: Schematic diagram of CRLF1 structure and damaged cartilage. IL-1β stimulates chondrocytes, leading to downregulated miR-8485 expression. MiR-8485 downregulation weakens its inhibition of CRLF1; CRLF1 expression is upregulated, activating the MAPK/ERK and PI3K/AKT signaling pathways, ultimately leading to chondrocyte damage and promoting OA progression. IGFBP7: Schematic diagram of IGFBP7 structure and damaged cartilage. IGFBP7-OT promotes osteoarthritis progression through the DNMT1/DNMT3a-IGFBP7 axis to upregulate IGFBP7. METTL3-mediated m6A modification of IGFBP7-OT upregulates the expression of IGFBP7-OT, subsequently reducing the binding of DNMT1/DNMT3a to the IGFBP7 promoter, leading to decreased DNA methylation levels in the IGFBP7 promoter and increased IGFBP7 transcription. The increase in IGFBP7 promotes chondrocyte apoptosis and accelerates the progression of osteoarthritis. S100A4: Schematic diagram of S100A4 structure and damaged cartilage. S100A4 binds to RAGE and stimulates the RAGE-mediated signaling cascade, resulting in increased production of MMP13. As an extracellular signaling molecule, S100A4 activates the pyk-2 and MAPK signaling pathways by binding to the RAGE receptor, thereby promoting NF-κB-mediated MMP-13 expression upregulation, ultimately leading to cartilage degradation. This signaling pathway is one of the crucial mechanisms in OA cartilage destruction. Created in BioRender. sun, Y. (2026) https://BioRender.com/tlwi72b**.**

The 11 co-DEFs that are upregulated in OA may serve as candidate biomarkers for OA following subsequent validation experiments. Among them, the factor MMP13 is very common with extensive studies conducted on it in the literature, so this review does not elaborate further. Additionally, there are more reports on ADAMTS4 and CRLF1, and some studies suggest associations between IGFBP7 and S100A4 factors and osteoarthritis. The remaining factors currently lack experimental articles in the OA field. ADAMTS4, which stands for ADAM metallopeptidase with thrombospondin type 1 motif 4, belongs to the Metzincin protease family. This protease is responsible for the degradation of major proteoglycans in cartilage [182,183]. De Groot et al. have demonstrated that ADAMTS4 is an effective COMP enzyme with a unique ability to cleave COMP, potentially playing a key role in regulating the structural integrity of cartilage ECM. ADAMTS4 inhibitors could be developed in the future as drugs to improve OA [184]. Yang et al. found that cysteine-rich angiogenic inducer 61 (Cyr61) promotes OA chondrocyte proliferation by inhibiting ADAMTS4 [185]. CRLF1, or cytokine receptor-like factor 1, is a soluble protein homologous to type I cytokine receptors. Existing studies have shown that CRLF1 is highly expressed in damaged human KOA and participates in OA development [186,187]. Xu et al. observed that CRLF1 significantly increases in an osteoarthritis model induced by destabilization of the medial meniscus (DMM) surgery and knockdown of CRLF1 inhibits apoptosis in primary chondrocytes [188]. Yang et al. found that miR-8485 inhibits the expression of CRLF1, thereby suppressing IL-1β-induced OA [189]. Inhibition of CRLF1 reduces IL-1β-induced cell viability impairment, apoptosis, inflammation, and extracellular matrix degradation [190]. IGFBP7, which stands for insulin-like growth factor binding protein 7, is a gene that encodes a member of the insulin-like growth factor binding protein family. Tang et al. discovered that m6A-modified IGFBP7-OT promotes osteoarthritis progression via regulation of the DNMT1/DNMT3a-IGFBP7 axis and provides a putative therapeutic target for osteoarthritis treatment [191]. S100A4, full name S100 calcium binding protein A4, is a member of the S100 protein family and regulates various cellular processes involved in cell cycle progression and differentiation. Yammani et al. first demonstrated that S100A4 binds to the receptor for advanced glycation end products (RAGE) and stimulates the RAGE-mediated signaling cascade, which leads to increased production of MMP13. This signaling pathway may contribute to the degradation of OA cartilage [192]. Numerous factors in the OA field remain insufficiently studied, and these unknown factors may serve as important breakthroughs for future research. A schematic diagram illustrating the structures of the above four factors and their promotion of OA progression is shown in Figure 5.

These ten genes, including ASPN and MATN3, exhibit distinct expression trends between KOA and HOA. Among them, four factors—COL2A1, COL10A1, CLEC3B, and SPARC—show opposite expression patterns in knee versus hip omics data, which may be attributable to the different pathogenic triggers and core driving mechanisms of the two diseases. The remaining six genes display two forms of inconsistency in their expression trends between knee and hip omics. The first is intra-omic inconsistency, meaning that within the same joint and same omic layer, different studies report opposing directions. This phenomenon is likely due to heterogeneity in control group composition—the control samples included in the analyses cover normal cartilage, cartilage from femoral neck fracture donors, relatively intact cartilage from OA joints, and lesioned cartilage—rather than to intrinsic biological differences between OA of the knee and hip joints. The second is cross-omic inconsistency, where the same gene shows opposing directions at the RNA and protein levels within the same joint, which may arise from platform differences and as-yet-unresolved factors at the post-transcriptional regulatory level. In summary, these candidate molecules identified on the basis of differentially expressed genes between KOA and HOA require rigorous validation through subsequent confirmatory studies and clinical research.

## 8. Limitations

This review has several limitations. First, the literature search was restricted to a single database (PubMed), which may have led to the omission of relevant studies and compromised the comprehensiveness of evidence inclusion. Second, the included studies exhibited heterogeneity in terms of sample sources and omics platforms. Additionally, due to the scarcity of healthy control samples, we expanded the definition of the control group to include multiple types of controls, such as normal cartilage, femoral neck fracture tissues, and intact cartilage regions from osteoarthritic joints. This strategy may have contributed to inconsistent expression trends of candidate factors across different studies. Third, most of the included studies focused on mechanistic explorations, and there is a lack of independent large-scale clinical cohorts for external validation of the candidate factors. Therefore, the clinical translational value of these factors remains to be further elucidated.

## 9. Conclusions

This review systematically delineates the commonalities and differences between KOA and HOA at the gene expression level, offering a novel perspective for a deeper understanding of the molecular mechanisms underlying joint-specific osteoarthritis. Furthermore, the candidate molecules compiled herein establish a solid data foundation for the future discovery of biomarkers and therapeutic targets, fully underscoring the academic contribution and translational potential of this review.

## Figures and Tables

**Table 1:** Differentially expressed genes common to both KOA and HOA summarized from the literature.

Sample Type	Species	Experimental Group	Omics Platform and Technology	Statistical Methods	co-DEFs		Year	References
					Upregulated in Both Hip and Knee	Downregulated in Both Hip and Knee		
Cartilage Tissue	Human	Female HOA (n = 9) vs. Female NOF (n = 10); Female KOA vs. Female NOF	Transcriptomics; Illumina Human HT-12 V3 whole-genome expression array; Agilent GeneSpring GX 11; qRT-PCR validation of 6 genes (TaqMan, ABI PRISM 7900HT, Thermo Fisher Scientific Inc., 168 Third Avenue, Waltham, MA 02451, USA)	Mann–Whitney U test (non-parametric); quantile normalization + median baseline transformation; ComBat batch correction; unsupervised hierarchical clustering (Euclidean + complete linkage); GSEA (permutation test); IPA pathway analysis (Fisher’s exact test); STRING PPI network; Pearson correlation (knee/hip concordance)	CCDC80, CHD9, SPTAN1, GPR125, COL13A1, GALNT1, GNG2, EXTL2, CYB5A, GRN, RAPH1, CAPS, TLE4, CTSO, SCARA3, FZD7, TUBB2A, COPZ2, INSC, ENAH, NCAM1, HTRA1, ATP1B1, ATRN, SERBP1, COL8A2, EML1, LPHN1, PKM2, C20orf82, CTSK, PDGFRL, ANTXR1, DIXDC1, ITGA11, RAMP3, LSP1, NUAK1, C18orf1, ZNF503, ANGPTL2, PDGFC, ARHGDIB, EPB41L2, SHC4, HSPB6, FAT, OMD, SLC35B2, COL5A1, CHST6, ISLR, CALM1, GNG11, RPESP, MAGED1, CLEC11A, TSPAN2, HCFC1R1, ACPL2, ECM2, SMOC1, FZD8, GLI3, KAL1, PCOLCE, OAF, GREM1, COL11A1, DKK1, COL5A2, CRIP1, MAMDC2, OGN, CTHRC1, MXRA5, CLEC3A, SPARC, COL2A1, SERTAD4, CRISPLD1	SOD2, GLRX, TMOD1, ADM, ANGPTL7, MARCH3, MT1M, ELL2, EFNA1, HIST1H1C, GYG1, HIG2, ISG20, CEBPD, BTG2, SLC39A14, GADD45A, CMAH, C10orf10, AVPI1, MTHFD2, NEBL, KIAA1622, GBP2, ERRFI1, GLUL, GALNTL2, BEX2, RRAGD, CHAC2, ALPK1, IFRD1, DDIT4L, CBLB, ZNF395, ITGA5, WDR33, WSB1, TRIB3, NUPR1, THADA, FLJ22639, MST150, BCL6, PYGL, FAM134A, HMGB2, ANKRA2, CLK1, CCNG2, RBM7, GABARAPL1, EGFLAM, GNPDA1, MKNK2, DHX40, ASS1, MXI1, RHOBTB3, SESTD1, C2orf56, WHSC1L1, ELP2, AKT3, CHD2, UBE2V2, C9orf72, RAB11FIP5, CLK3, CCDC45, C17orf48, CNOT8, ANKRD28 ZFAND5, CTH, JAK2, AASS	2012	[86]
Cartilage Tissue	Human	HOA (n = 17) vs. NC (n = 17); KOA (n = 14) vs. NC (n = 14)	Transcriptomics; Illumina Infinium HumanMethylation450 BeadChip	PCA; linear mixed models for differential CpGs; sliding-window DMR algorithm; R v3.0.2	LOC375295, PITX1, DLX5, GMDS, SCNN1A, HOXB2, HOXC8, MEIS2, SGK, IRX3, HOXC6	Not mentioned	2014	[87]
Cartilage Tissue	Human	Damage dcartilage in HOA (n = 10) vs. NC (n = 6); Damage dcartilage in KOA vs. NC	Transcriptomics; Illumina Genome Analyzer Iix; Illumina HiSeq 2000 (Illumina, Inc., San Diego, California, USA.)	DESeq2; RUVg removal of unwanted variation; PCA	RP1-125N5.2, LINC00885, RP11-161M6.2, RP11-449D8.1, PSMG3-AS1, AC159540.1, AP006621.5, LINC01089, RP11-63G10.4, LINC00342	AC144831.1	2019	[88]
Cartilage Tissue	Human	HOA vs. NC; KOA vs. NC	Proteomics; nanoLC-LTQ-Orbitrap Velos Pro (Thermo Fisher Scientific, Waltham, Massachusetts, USA)	One-way ANOVA and paired Student’s *t*-test (GraphPad Prism 5.0); multivariate regression (joint site, disease state, cartilage depth; JMP Pro 11.2); hierarchical clustering (Cluster 3.0 + JAVA Treeview); MRM peak-area relative quantification	TNXB, FN1, COL6A3, COL12A1, COL2A1, TNC, C3, THBS1, COMP, COL3A1, HSPG2, ALB, ACAN, CILP, CILP2, COL1A1, CFH, COL1A2, FBN1, TF, C4B, C4A, VIM, THBS4, KRT1, FGB, HAPLN1, COL6A2, LTF, COL6A1, PKM, MATN3, KRT10, LMNA, FGG, TGFBI, BGN, PRELP, CLU, FGA, MEGE8, KRT9, PLG, ANXA1, MMP3, ANXA2, ABI3BP, COL11A2, KAL1, DCN, SRPX2, GSN, MMP2, HHIPL2, CFB, GAPDH, IGHG1, ENO1, VIT, AEBP1, OGN, PGK1, ASPN, HSPA5, AHNAK, FMOD, LUM, PCOLCE, KRT2, HP, FBLN1, PRG4, POSTN, CHAD, HBD, SERPINF1, HTRA1, ALDOA, SMOC2, F2, KRT6A, CKM, KRT16, COL5A2, HBB, IGHG2, PCOLCE2, IGHA1, VCAN, HPX, A2M, IGHG3, CLEC3A, LDHA, ACTB, SERPINE2, KRT5, KRT14, APOE, C9, HSP9OB1, PDIA3, LYZ, IGHG4, FGFBP2, VTN, CLEC3B, SERPINA1, KRT6B, ACTC1, HSPA8, CHI3L1, ITIH1, CCDC80, BPI, ANGPTL2, GDF10, CP, EFEMP1, APOA4, ITIH2, S0D3, ANG, PLA2G2A, FHL1, HSPA1B, PRDX1, CA3, APOA1, SERPINA3, MSN, MMP9, C1R, EDIL3, IGKC, HBA2, APCS, PPIA, PPIB, EEF1A1, IGHA2, COL11A1, COL5A1, ANXA4, ITIH6, C8B, RNASE4, PEBP1, C1QB, PPBP, AMBP, AK1, HSPA1L, HIST1H2BK, HIST1H2BJ, KRT13, PEN1, ANXA5, C8G, FRZB, BLVRB, CFD, TUBA1B, MNDA, CA2, COL9A1, FKBP3, SERPINH1, GC, IGHM, C8A, TKT, COL16A1, MEI2, PRDX2, MAMDC2, IGLC2 (trends not mentioned)		2017	[89]
Chondrocyte	Human	Degenerated cartilage in HOA vs. Intact cartilage in HOA	Proteomics; UK Biobank genotypes	Case–control association analysis (OR with 95% CI)	GDF5, MCF2L, FTO, SUPT3H, CDC5L, SMAD3, GLT8D1, DVWA, HLA-DQB1, BTNL2, DUS4L, TP63, COG5, DOT1L, NCOA3, FILIP1, SENP6, KLHDC5, PTHLH, ASTN2, CHST11 (trends not mentioned)		2018	[90]

NOF, neck of femur fracture.

**Table 3:** Differentially expressed proteins in the KOA proteome (top 10 DEPs listed).

Sample Type	Species	Experimental Group	Omics Platforms	Statistical Methods	Top 10 DEPs		Year	References
					Upregulated	Downregulated		
Cartilage Tissue	Human	OA (n = 3, Male = 1 Female = 2) vs. NC (n = 3, Male = 2 Female = 1)	2-DE/MS	Not mentioned	ASPIC, CILP, COMP, YKL-39, YKL-40, MMP1, MMP2, MMP3, TIMP1, TIMP2 (trends not mentioned)		2004	[138]
Cartilage Tissue	Human	OA vs. NC	2-DE/MS	Student’s *t*-test	ANNX-I, PRDX3, Zn-RF, FR, TUB, ADK, PEBP, SODM, ENOA, KPYM	Not mentioned	2008	[139]
Cartilage Tissue	Human	Weight-bearing area of OA (n = 5) vs. non-weight-bearing region of OA (n = 5)	HPLC-MS	Wilcoxon rank-sum test	CPN2, C1OB, SERP1NA3, SERP1NA10, SERP1NA4, MMP19, FETUB, SERP1NG1, AGT, SSR1	SRPX2, ARF3, CRYZ, HSPG2, ANOS1, IGF1, SMOC2, GBE1, ANXA4, CH1	2023	[111]
Cartilage Tissue	Human	OA (no NC)	LC-MS/MS	Not mentioned	ACAN, CILP1, HAPLN1, FN1, COMP, COL6A1, FMOD, BGN, CHAD, OGN (trends not mentioned)		2006	[140]
Cartilage Tissue	Human	OA (n = 15, Male = 6 Female = 9) vs. NC (n = 11, Male = 6 Female = 5)	LC-MS/MS	Student’s *t*-test	LAMP1, PDXDC1, RUVBL1, SPCS3, SNRPD3, BAP18, ESYT1, RPL13A, IDH2, LTBP2	AGRIN, ANGPTL5, SLC1A5, AGRN, EPHX2, ATRN, CYRIB, NHP2, IGHV3-72, SPON2	2025	[141]
Cartilage Tissue	Human	OA Cartilage after KJD vs. NC	LC-MS/MS	Student’s *t*-test	SNRPD2, RPL19, CRYAB, NID1, OGN, RPL26, LRPB, ANOS1, RPS2, CAVIN1	MYO1C, ATP5F1A, MDH2, C1QA, PGK2, SERPINC1, SNRPB, SOD3, CHAD, SELENBP1	2024	[142]
Cartilage Tissue	Human	OA with degenerated cartilage (G4) vs. relatively intact cartilage in OA (G1)	LA-ICP-MS	Independent samples *t*-test	GAPDH, ACTB, SOD1, ANXA2, ALB, ENO1, S100A7, FN1, RPS27A, HSPAB (trends not mentioned)		2022	[143]
Cartilage Tissue	Human	Unfixed OA cartilage vs. fixed OA cartilage	LC-MS/MS (label-free, limma)	Paired *t*-tests	HMOX1, TNFRSF11B, SQLE, SLC3A2, FDFT1, DHCB24, SQSTM1, CYP51A1, SLC39A14, TNFRSF10B	FOS, FGFR2, FOXO3, GABARAPL2, IFITM1, CTNNB1, STAT3, RXRA, CALML3, FOXO1	2025	[144]
Chondrocyte	Human	OA-damaged cartilage (OAOA) (n = 6) vs. NC (NoNo) (n = 7)	2-DE/MS;	Mann–Whitney U; Wilcoxon paired test	Vimentin, eIF5A, HEXB, STOML2, HSC70, HSP70-1, PDIA3, VDAC2, ANXA1, ENO1	CUTA, SOD1, PARK7, DJ-1, PRDX6, FABP, CSTB, NAE1, UBA3	2007	[145]
Chondrocyte	Human	H_2_O_2_ treatment of OA (n = 14) vs. HA/H_2_O_2_ treatment of OA (n = 14) Male = 1 Female = 13	2-DE/MS;	ANOVA + Tukey HSD; paired *t*-test	TALDO1, SELENBP1, RAN	HSPE1, RAB14, FSCN1, PPP2R1A, EEF2, RPLP0	2014	[146]
Chondrocyte	Human	OA (n = 10, Male = 1 Female = 9) vs. NC (n = 6, Male = 3 Female = 3)	LC-MS/MS	*t*-test	TG2, MVP, MYOF, CTSB, PPDX2, PLS3, GSTP1	ACAN, APCS, COL2A1, PLA2G2A	2015	[147]
Chondrocyte	Human	OA with degenerative cartilage vs. OA with intact cartilage	LC-MS/MS	Paired *t*-test	UNC45B, TNMD, WNT11, HMGN5, CLC, HMGN3, KRT3, CENPF, COL23A1, GRM7 (trends not mentioned)		2017	[128]
Chondrocyte	Human	OA-damaged cartilage (G4) (n = 20) vs. intact smooth cartilage in OA (G1) (n = 20) Male = 8 Female = 12	LC-MS/MS	Paired *t*-test, two-way ANOVA + Tukey post hoc	MT1X, MT1G, YBX3, TNPO2, SERPINB3, ECI1, RBMXL2, CYRIB, CSNK2B, TGM3	HSPA6, KRT13, YOD1, IFITM2, VAMP2, NHLRC3, RBP1, PABPN1, SEMG2, IGHV1OR15-1	2022	[129]
Chondrocyte	Human	miR-143-3p-transfected OA vs. OA	LC-MS/MS	*t*-test	RTCB, PSMA7, SUCLG1, RPS14, DPM3, PCNA, CZIB, PPP2R1A, FBL, ARL3	RPL24, DIABLO, PABPC4, CYFIP1, SRSF3, FLOT1, TOM1, CSTB, GET3, RPL28	2023	[148]
Chondrocyte	Human	OA (n = 10) vs. NC (n = 6)	1-DE/RP-LC-MS	Student’s *t*-test	PRDX2, AP2A2, RAC1, CSNK2A1, STAT, HNRNPL, CD44, RPLPO, PHB2, PALLD	ALDOA, PRDX1, EDIL3, TXNDC5, LMAN2, PSAT1, COL6A2, UGP2, AARS1, COL11A1	2021	[149]
Chondrocyte	Human	OA-damaged cartilage vs. intact smooth cartilage in OA	MS	Student’s *t*-test; ANOVA	THBS, SOD2, RCN3, PLBD2, PFDN2, PDIA3, RAB1A, AKAP12, FLNA, PGAM1	CRYAB, NME2, ALDOC, COL14A1, HSPA8, HSPB6, HSPB1, ACADVL, NUPR1, HSPA1A	2024	[150]
Cartilage Tissue	Bama Mini-Pig	Female OA vs. Female NC	LC-MS/MS	Fisher’s exact test	KRAS, HRAS, NRAS, MAPK1, PIK3CA, COX5A, COX6C, APP, PSEN1, MAPT		2020	[151]
Chondrocyte	Mice	OA vs. Silk fibroin protein-treated OA cartilage	LC-MS/MS	ANOVA + independent *t*-test	Not mentioned	PGAM1, UBC, TPIS, COCA1, COAA1, RABIA, POSTN, PROF1, LEG1, Q9ZOI9	2024	[152]

KJD, knee joint distraction treatment; MS, mass spectrometry; 2-DE/MS, two-dimensional gel electrophoresis–mass spectrometry; MS/MS, tandem mass spectrometry; LC-MS/MS, liquid chromatography–tandem mass spectrometry; LA-ICP-MS, laser ablation inductively coupled plasma mass spectrometry; HPLC-MS, high-performance liquid chromatography; 1-DE/RP-LC, nano-reverse phase (RP) LC; H2O2, hydrogen peroxide; HA, hyaluronic acid; miR-143-3p, microRNA 143.

## Data Availability

The original contributions presented in this study are included in the article/Supplementary Materials. Further inquiries can be directed to the corresponding authors.

## Supplementary Materials

The following supporting information can be downloaded at: https://www.mdpi.com/article/10.3390/biom16091363/s1, Table S1: PubMed advanced search records; Table S2: Omics literature on knee and hip osteoarthritis; Table S3: Trends of the 31 factors; Table S4: Quality assessment form.

## Abbreviations

The following abbreviations are used in this manuscript:

OA	Osteoarthritis
DEGs	Differentially expressed genes
DEPs	Differentially expressed proteins
DEFs	Differentially expressed factors
Co-DEGs	Common differentially expressed genes
Co-DEPs	Common differentially expressed proteins
Co-DEFs	Common differentially expressed factors
KOA	Knee osteoarthritis
HOA	Hip osteoarthritis
CCDC80	Coiled-coil domain containing 80
HTRA1	HtrA serine peptidase 1 gene
ACAN	Aggrecan
COMP	Cartilage oligomeric matrix protein
CILP2	Cartilage intermediate layer protein 2
CHRDL2	Chordin-like 2
ANGPTL2	Angiopoietin-like 2
ADAMTS4	ADAM metallopeptidase with thrombospondin type 1 motif 4
CRLF1	Cytokine receptor-like factor 1,
IGFBP7	Insulin-like growth factor binding protein 7
S100A4	S100 calcium binding protein A4
2-DE	Two-dimensional gel electrophoresis
LC-MS/MS	Liquid chromatography–tandem mass spectrometry
LC-MS	Liquid chromatography–mass spectrometry
NOF	Neck of femur fracture
DMRs	Differentially methylated regions
WISP-1	WNT1 inducible signaling pathway protein 1
AQP-1	Aquaporin 1
DNER	Delta/notch-like EGF repeat containing
KBD	Kashin–Beck disease
GEO	Gene Expression Omnibus
NCBI	National Center for Biotechnology Information
TWAS	Transcriptome-wide association study
CGSEA	Cluster-based gene set enrichment analysis
SIFK	Subchondral incomplete fracture of the knee joint
preHTC	Prehypertrophic chondrocytes
KJD	Knee joint distraction
ACLR	Anterior cruciate ligament reconstruction
FAI	Femoroacetabular impingement
RPLC	Reversed-phase liquid chromatography
GAG	Glycosaminoglycan
Cyr61	Cysteine-rich angiogenic inducer 61

## References

[1] Yang Z., Hou N., Yang B., Lin W., Cai G., Zheng L., Cheung C.H.P., Wang Y., Chen H., Wang Y. (2025). DHA Laden Liposome Incorporated HA-Menthol-CD Host-Guest Hydrogels as Delivery System for Osteoarthritis Treatment. Int. J. Biol. Macromol..

[2] Samvelyan H.J., Hughes D., Stevens C., Staines K.A. (2021). Models of Osteoarthritis: Relevance and New Insights. Calcif. Tissue Int..

[3] Wu Z., Xu H., Qiao B., Zhu Y., Yuan R., Wu Z., Li S., Li J., Xu Y., Dong W. (2025). Yiqi Xugu HeJi Restores Cartilage Metabolic Homeostasis via AKT1-Thr473 Activation in Osteoarthritis. Phytomedicine.

[4] Gu J., Rao W., Huo S., Fan T., Qiu M., Zhu H., Chen D., Sheng X. (2022). MicroRNAs and Long Non-Coding RNAs in Cartilage Homeostasis and Osteoarthritis. Front. Cell Dev. Biol..

[5] Liu Y., Li M., Yin Z., Zhou S., Qiu Y. (2020). SUMO-Modified Bone Marrow Mesenchymal Stem Cells Promoted the Repair of Articular Cartilage in Rats. Cell Biol. Int..

[6] Liu X., Gao F., Wang W., Yan J. (2020). Expression of MiR-204 in Patients with Osteoarthritis and Its Damage to Chondrocytes. J. Musculoskelet. Neuronal Interact..

[7] Veronese N., Trevisan C., De Rui M., Bolzetta F., Maggi S., Zambon S., Musacchio E., Sartori L., Perissinotto E., Crepaldi G. (2016). Association of Osteoarthritis With Increased Risk of Cardiovascular Diseases in the Elderly: Findings From the Progetto Veneto Anziano Study Cohort. Arthritis Rheumatol..

[8] Hall A.J., Stubbs B., Mamas M.A., Myint P.K., Smith T.O. (2016). Association between Osteoarthritis and Cardiovascular Disease: Systematic Review and Meta-Analysis. Eur. J. Prev. Cardiol..

[9] Sun W., Ai D., Yao Y., Ren K., Lu J., Sun H., Wu X., Jiang Q. (2022). The Application of Caprini Risk Assessment Model in Evaluation of Deep Vein Thrombosis for Patients with End-Stage Osteoarthritis before Arthroplasty. BMC Musculoskelet. Disord..

[10] Hon K.P.B., Shen Xuanrong M., Poon K.B. (2025). Epidemiological Study on the Prevalence and Severity of Knee Osteoarthritis in Geriatric Patients With Fragility Fractures of the Hip. Cureus.

[11] Mekariya K., Vanitcharoenkul E., Chotiyarnwong P., Adulkasem N., Unnanuntana A. (2025). High Prevalence of Symptomatic Knee Osteoarthritis Among Patients Who Have Fragility Hip Fractures. J. Arthroplast..

[12] Courties A., Kouki I., Soliman N., Mathieu S., Sellam J. (2024). Osteoarthritis Year in Review 2024: Epidemiology and Therapy. Osteoarthr. Cartil..

[13] Xiong Z., Peng G., Deng J., Liu M., Ning X., Zhuang Y., Yang H., Sun H. (2024). Therapeutic Targets and Potential Delivery Systems of Melatonin in Osteoarthritis. Front. Immunol..

[14] Li H.-Z., Liang X.-Z., Sun Y.-Q., Jia H.-F., Li J.-C., Li G. (2024). Global, Regional, and National Burdens of Osteoarthritis from 1990 to 2021: Findings from the 2021 Global Burden of Disease Study. Front. Med..

[15] Cigercioglu N.B., Bazancir-Apaydin Z., Apaydin H., Baltaci G., Guney-Deniz H. (2024). Differences in Ankle and Knee Muscle Architecture and Plantar Pressure Distribution among Women with Knee Osteoarthritis. J. Foot Ankle Res..

[16] Feng C., Zhang Y., Li W., Liu Y., Duan C., Ma J., Wang Y., Zhuang R., Ding Y. (2023). Identification of CaMK4 as a Sex-Difference-Related Gene in Knee Osteoarthritis. Ann. Transl. Med..

[17] Hunter D.J., March L., Chew M. (2020). Osteoarthritis in 2020 and beyond: A Lancet Commission. Lancet.

[18] Hana S., Aicha B.T., Selim D., Ines M., Rawdha T. (2018). Clinical and Radiographic Features of Knee Osteoarthritis of Elderly Patients. Curr. Rheumatol. Rev..

[19] Shu Z., Miao X., Tang T., Zhan P., Zeng L., Jiang Y. (2020). The GSK-3β/Β-catenin Signaling Pathway Is Involved in HMGB1-induced Chondrocyte Apoptosis and Cartilage Matrix Degradation. Int. J. Mol. Med..

[20] Yang T., Jia C., Wang G., Zhang W., Zhu B., Li X., Chen L., Li Y., Teng Z. (2025). Uncovering Causal Genetic Mediators Linking Redefined Obesity to Osteoarthritis: Multidimensional Analysis from Population to Genetic Mechanisms. Osteoarthr. Cartil..

[21] Weng Q., Chen Q., Jiang T., Zhang Y., Zhang W., Doherty M., Xie J., Liu K., Li J., Yang T. (2024). Global Burden of Early-Onset Osteoarthritis, 1990–2019: Results from the Global Burden of Disease Study 2019. Ann. Rheum. Dis..

[22] Laires P.A., Canhão H., Rodrigues A.M., Eusébio M., Gouveia M., Branco J.C. (2018). The Impact of Osteoarthritis on Early Exit from Work: Results from a Population-Based Study. BMC Public Health.

[23] Rumalla K.C., Chandrupatla S.R., Singh J.A. (2025). Intersectional Racial, Ethnic, and Sex-Based Disparities in Length of Stay after Total Hip and Knee Arthroplasty: An Analysis of National Data. Osteoarthr. Cartil..

[24] Leifer V.P., Katz J.N., Losina E. (2022). The Burden of OA-Health Services and Economics. Osteoarthr. Cartil..

[25] Lv Z., Wang Z., Chen D., Shi D. (2024). Advances in Osteoarthritis Research: From Diagnosis, Treatment to Mechanism Studies. J. Orthop. Transl..

[26] Wu J., Zhang Z., Ma X., Liu X. (2023). Advances in Research on the Regulatory Roles of LncRNAs in Osteoarthritic Cartilage. Biomolecules.

[27] Ma X., Zhang Z., Shen M., Ma Y., Li R., Jin X., Gao L., Wang Z. (2021). Changes of Type II Collagenase Biomarkers on IL-1β-Induced Rat Articular Chondrocytes. Exp. Ther. Med..

[28] Huang D., Feng Z.G., Hu R., Li Y.H., Liang L.S., Liu Q., Liu T.H., Ma K., Wang L., Wang S.L. (2024). Chinese guidelines for diagnosis and treatment of osteoarthritis (2024 edition). Chin. J. Painol..

[29] Agrawal N. (2025). A Comprehensive Review on the Advancements of Dual COX-2/5-LOX Inhibitors as Anti-Inflammatory Drugs. Chem. Biol. Drug Des..

[30] Labarre K.W., Zimmermann G. (2025). Long-Term Effects of Infrapatellar Fat Pad SVF Infiltration in Knee Osteoarthritis Management: A Prospective Cohort Study. Bone Rep..

[31] Qin Y., Di J., Guo Z., Chen S., Xiang C. (2025). Association of Organs-Crosstalk with the Pathogenesis of Osteoarthritis: Cartilage as a Key Player. Front. Endocrinol..

[32] Dorraki M., Muratovic D., Fouladzadeh A., Verjans J.W., Allison A., Findlay D.M., Abbott D. (2022). Hip Osteoarthritis: A Novel Network Analysis of Subchondral Trabecular Bone Structures. PNAS Nexus.

[33] Yin W.-J., Xu H.-T., Sheng J.-G., An Z.-Q., Guo S.-C., Xie X.-T., Zhang C.-Q. (2016). Advantages of Pure Platelet-Rich Plasma Compared with Leukocyte- and Platelet-Rich Plasma in Treating Rabbit Knee Osteoarthritis. Med. Sci. Monit..

[34] Vincent T.L., Miller R.E. (2024). Molecular Pathogenesis of OA Pain: Past, Present, and Future. Osteoarthr. Cartil..

[35] Hurley M., Dickson K., Hallett R., Grant R., Hauari H., Walsh N., Stansfield C., Oliver S. (2018). Exercise Interventions and Patient Beliefs for People with Hip, Knee or Hip and Knee Osteoarthritis: A Mixed Methods Review. Cochrane Database Syst. Rev..

[36] Fransen M., McConnell S., Harmer A.R., Van Der Esch M., Simic M., Bennell K.L. (2015). Exercise for Osteoarthritis of the Knee. Cochrane Database Syst. Rev..

[37] Kroon F.P., Van Der Burg L.R., Buchbinder R., Osborne R.H., Johnston R.V., Pitt V. (2014). Self-Management Education Programmes for Osteoarthritis. Cochrane Database Syst. Rev..

[38] Lee K.-M., Guo J. (2010). Kinematic and Dynamic Analysis of an Anatomically Based Knee Joint. J. Biomech..

[39] Altai Z., Hayford C.F., Phillips A.T.M., Moran J., Zhai X., Liew B.X.W. (2024). Lower Limb Joint Loading during High-Impact Activities: Implication for Bone Health. JBMR Plus.

[40] Bastick A.N., Belo J.N., Runhaar J., Bierma-Zeinstra S.M.A. (2015). What Are the Prognostic Factors for Radiographic Progression of Knee Osteoarthritis? A Meta-Analysis. Clin. Orthop. Relat. Res..

[41] Gierisch J.M., Myers E.R., Schmit K.M., McCrory D.C., Coeytaux R.R., Crowley M.J., Chatterjee R., Kendrick A.S., Sanders G.D. (2014). Prioritization of Patient-Centered Comparative Effectiveness Research for Osteoarthritis. Ann. Intern. Med..

[42] Gooberman-Hill R., French M., Dieppe P., Hawker G. (2009). Expressing Pain and Fatigue: A New Method of Analysis to Explore Differences in Osteoarthritis Experience. Arthritis Rheum..

[43] Poulsen E., Overgaard S., Vestergaard J.T., Christensen H.W., Hartvigsen J. (2016). Pain Distribution in Primary Care Patients with Hip Osteoarthritis. Fam. Pract..

[44] Ye M., Lin Y., Pan S., Wang Z.-W., Zhu X. (2021). Applications of Multi-Omics Approaches for Exploring the Molecular Mechanism of Ovarian Carcinogenesis. Front. Oncol..

[45] Xu M., Wei Z., Wang L., Liang W., Gao F., Sang S., Zhang R. (2025). Advances in Multi-Omics and Therapeutic Studies of Henoch-Schönlein Purpura. Ital. J. Pediatr..

[46] Kuang J., Lin Y., Wang L., Yan Z., Wei J., Du J., Li Z. (2024). Effects of PEF on Cell and Transcriptomic of Escherichia Coli. Microorganisms.

[47] McGettigan P.A. (2013). Transcriptomics in the RNA-Seq Era. Curr. Opin. Chem. Biol..

[48] Lowe R., Shirley N., Bleackley M., Dolan S., Shafee T. (2017). Transcriptomics Technologies. PLoS Comput. Biol..

[49] Dong Z., Chen Y. (2013). Transcriptomics: Advances and Approaches. Sci. China Life Sci..

[50] França L.T.C., Carrilho E., Kist T.B.L. (2002). A Review of DNA Sequencing Techniques. Q. Rev. Biophys..

[51] Giani A.M., Gallo G.R., Gianfranceschi L., Formenti G. (2020). Long Walk to Genomics: History and Current Approaches to Genome Sequencing and Assembly. Comput. Struct. Biotechnol. J..

[52] AlEisa H.N., Hamad S., Elhadad A. (2022). K-Mer Spectrum-Based Error Correction Algorithm for Next-Generation Sequencing Data. Comput. Intell. Neurosci..

[53] Wang Z., Gerstein M., Snyder M. (2009). RNA-Seq: A Revolutionary Tool for Transcriptomics. Nat. Rev. Genet..

[54] Prieto-Vila M., Yamamoto Y., Takahashi R., Ochiya T., Santra T.S., Tseng F.-G. (2018). Single-Cell Transcriptomics. Handbook of Single Cell Technologies.

[55] Rodriques S.G., Stickels R.R., Goeva A., Martin C.A., Murray E., Vanderburg C.R., Welch J., Chen L.M., Chen F., Macosko E.Z. (2019). Slide-Seq: A Scalable Technology for Measuring Genome-Wide Expression at High Spatial Resolution. Science.

[56] Oliveira M.F.D., Romero J.P., Chung M., Williams S.R., Gottscho A.D., Gupta A., Pilipauskas S.E., Mohabbat S., Raman N., Sukovich D.J. (2025). High-Definition Spatial Transcriptomic Profiling of Immune Cell Populations in Colorectal Cancer. Nat. Genet..

[57] Schott M., León-Periñán D., Splendiani E., Strenger L., Licha J.R., Pentimalli T.M., Schallenberg S., Alles J., Samut Tagliaferro S., Boltengagen A. (2024). Open-ST: High-Resolution Spatial Transcriptomics in 3D. Cell.

[58] Zambon M., Mantica F., Dias M., Frazer J., Irimia M. (2025). Evolution of Comparative Transcriptomics: Biological Scales, Phylogenetic Spans, and Modeling Frameworks. Curr. Opin. Genet. Dev..

[59] Aslam B., Basit M., Nisar M.A., Khurshid M., Rasool M.H. (2017). Proteomics: Technologies and Their Applications. J. Chromatogr. Sci..

[60] O’Lone C.E., Juhász A., Nye-Wood M., Dunn H., Moody D., Ral J.-P., Colgrave M.L. (2023). Proteomic Exploration Reveals a Metabolic Rerouting Due to Low Oxygen during Controlled Germination of Malting Barley (*Hordeum vulgare* L.). Front. Plant Sci..

[61] la Marca G., Carling R.S., Moat S.J., Yahyaoui R., Ranieri E., Bonham J.R., Schielen P.C.J.I. (2023). Current State and Innovations in Newborn Screening: Continuing to Do Good and Avoid Harm. Int. J. Neonatal Screen..

[62] Pandey A., Mann M. (2000). Proteomics to Study Genes and Genomes. Nature.

[63] Park J., Sim H., Lee E.H., Kim B.S., Chung J.-W., Ha Y.-S., Kwon T.G., Lee S., Lee J.N. (2024). Comparative Proteomics of CcRCC Cell Lines to Identify Kidney Cancer Progression Factors. Cancer Genom. Proteom..

[64] Indovina P., Marcelli E., Maranta P., Tarro G. (2011). Lung Cancer Proteomics: Recent Advances in Biomarker Discovery. Int. J. Proteom..

[65] Teraiya M., Perreault H., Chen V.C. (2023). An Overview of Glioblastoma Multiforme and Temozolomide Resistance: Can LC-MS-Based Proteomics Reveal the Fundamental Mechanism of Temozolomide Resistance?. Front. Oncol..

[66] Di Tucci C., Muzii L. (2023). Chronic Pelvic Pain, Vulvar Pain Disorders, and Proteomics Profiles: New Discoveries, New Hopes. Biomedicines.

[67] Corana M., Baima G., Iaderosa G., Franco F., Zhang J., Berta G.N., Romano F., Aimetti M. (2025). Salivary Proteomics for Detecting Novel Biomarkers of Periodontitis: A Systematic Review. J. Periodontal Res..

[68] Görg A., Weiss W., Dunn M.J. (2004). Current Two-dimensional Electrophoresis Technology for Proteomics. Proteomics.

[69] Lee P.Y., Saraygord-Afshari N., Low T.Y. (2020). The Evolution of Two-Dimensional Gel Electrophoresis—From Proteomics to Emerging Alternative Applications. J. Chromatogr. A.

[70] Shi F., Zhang X., Wang Z., Wang X., Zou C. (2024). Unveiling Molecular Mechanisms of Pepper Resistance to Phytophthora Capsici through Grafting Using ITRAQ-Based Proteomic Analysis. Sci. Rep..

[71] Uitto P.M., Lance B.K., Wood G.R., Sherman J., Baker M.S., Molloy M.P. (2007). Comparing SILAC and Two-Dimensional Gel Electrophoresis Image Analysis for Profiling Urokinase Plasminogen Activator Signaling in Ovarian Cancer Cells. J. Proteome Res..

[72] Lu Z., Han C., Li C., Deng K., Shan Z., Chen S., Yang H., Yang Y., Yang Z., Wang H. (2025). Temporal Proteomic Profiling via 4D-DIA Reveals Early Defense Mechanisms and Core Resistance Determinants in Soybean against Phakopsora Pachyrhizi. Stress Biol..

[73] Liu T., Qian W.-J., Camp D.G., Smith R.D. (2007). The Use of a Quantitative Cysteinyl-Peptide Enrichment Technology for High-Throughput Quantitative Proteomics. Methods Mol. Biol..

[74] Gillenwater L.A., Pratte K.A., Hobbs B.D., Cho M.H., Zhuang Y., Halper-Stromberg E., Cruickshank-Quinn C., Reisdorph N., Petrache I., Labaki W.W. (2020). Plasma Metabolomic Signatures of Chronic Obstructive Pulmonary Disease and the Impact of Genetic Variants on Phenotype-Driven Modules. Netw. Syst. Med..

[75] Zhang W., Zhong T., Chen Y. (2017). LC-MS/MS-Based Targeted Proteomics Quantitatively Detects the Interaction between P53 and MDM2 in Breast Cancer. J. Proteom..

[76] Kulyyassov A., Fresnais M., Longuespée R. (2021). Targeted Liquid Chromatography-tandem Mass Spectrometry Analysis of Proteins: Basic Principles, Applications, and Perspectives. Proteomics.

[77] Hewel J.A., Liu J., Onishi K., Fong V., Chandran S., Olsen J.B., Pogoutse O., Schutkowski M., Wenschuh H., Winkler D.F.H. (2010). Synthetic Peptide Arrays for Pathway-Level Protein Monitoring by Liquid Chromatography-Tandem Mass Spectrometry. Mol. Cell. Proteom..

[78] Hansen K.C., Schmitt-Ulms G., Chalkley R.J., Hirsch J., Baldwin M.A., Burlingame A.L. (2003). Mass Spectrometric Analysis of Protein Mixtures at Low Levels Using Cleavable 13C-Isotope-Coded Affinity Tag and Multidimensional Chromatography. Mol. Cell. Proteom..

[79] Adkins J.N., Varnum S.M., Auberry K.J., Moore R.J., Angell N.H., Smith R.D., Springer D.L., Pounds J.G. (2002). Toward a Human Blood Serum Proteome. Mol. Cell. Proteom..

[80] Chen Y., Liu Y., Gao X. (2021). The Application of Single-Cell Technologies in Cardiovascular Research. Front. Cell Dev. Biol..

[81] Taniguchi Y. (2015). Genome-Wide Analysis of Protein and MRNA Copy Numbers in Single Escherichia Coli Cells with Single-Molecule Sensitivity. Methods Mol. Biol..

[82] Ren J., Li H.-W., Chen L., Zhang M., Liu Y.-X., Zhang B.-W., Xu R., Miao Y.-Y., Xu X.-M., Hua X. (2023). Mass Spectrometry Imaging-Based Single-Cell Lipidomics Profiles Metabolic Signatures of Heart Failure. Research.

[83] Su P.-R., Chien M.-P. (2025). Functional Single-Cell Proteomics: Technology and Biological Applications. Methods Mol. Biol..

[84] Guo T., Steen J.A., Mann M. (2025). Mass-Spectrometry-Based Proteomics: From Single Cells to Clinical Applications. Nature.

[85] Qian W.-J., Jacobs J.M., Liu T., Camp D.G., Smith R.D. (2006). Advances and Challenges in Liquid Chromatography-Mass Spectrometry-Based Proteomics Profiling for Clinical Applications. Mol. Cell. Proteom..

[86] Xu Y., Barter M.J., Swan D.C., Rankin K.S., Rowan A.D., Santibanez-Koref M., Loughlin J., Young D.A. (2012). Identification of the Pathogenic Pathways in Osteoarthritic Hip Cartilage: Commonality and Discord between Hip and Knee OA. Osteoarthr. Cartil..

[87] Den Hollander W., Ramos Y.F.M., Bos S.D., Bomer N., Van Der Breggen R., Lakenberg N., De Dijcker W.J., Duijnisveld B.J., Slagboom P.E., Nelissen R.G.H.H. (2014). Knee and Hip Articular Cartilage Have Distinct Epigenomic Landscapes: Implications for Future Cartilage Regeneration Approaches. Ann. Rheum. Dis..

[88] Ajekigbe B., Cheung K., Xu Y., Skelton A.J., Panagiotopoulos A., Soul J., Hardingham T.E., Deehan D.J., Barter M.J., Young D.A. (2019). Identification of Long Non-Coding RNAs Expressed in Knee and Hip Osteoarthritic Cartilage. Osteoarthr. Cartil..

[89] Hsueh M.-F., Khabut A., Kjellström S., Önnerfjord P., Kraus V.B. (2016). Elucidating the Molecular Composition of Cartilage by Proteomics. J. Proteome Res..

[90] Casalone E., Tachmazidou I., Zengini E., Hatzikotoulas K., Hackinger S., Suveges D., Steinberg J., Rayner N.W., Wilkinson J.M., Panoutsopoulou K. (2018). A Novel Variant in GLIS3 Is Associated with Osteoarthritis. Ann. Rheum. Dis..

[91] Li J., Wan T., Liu C., Liu H., Ke D., Li L. (2023). ANGPTL2 Aggravates LPS-Induced Septic Cardiomyopathy via NLRP3-Mediated Inflammasome in a DUSP1-Dependent Pathway. Int. Immunopharmacol..

[92] Endo M., Nakano M., Kadomatsu T., Fukuhara S., Kuroda H., Mikami S., Hato T., Aoi J., Horiguchi H., Miyata K. (2012). Tumor Cell–Derived Angiopoietin-like Protein ANGPTL2 Is a Critical Driver of Metastasis. Cancer Res..

[93] Shan W., Cheng C., Huang W., Ding Z., Luo S., Cui G., Lu W., Liu F., Xu J., He W. (2019). Angiopoietin-like 2 Upregulation Promotes Human Chondrocyte Injury via NF-ΚB and P38/MAPK Signaling Pathway. J. Bone Miner. Metab..

[94] Xiao Q., Li Y., Cai B., Huang X., Fang L., Liang F., Chen L., Xu K., Zhang W., Wang X. (2025). CCDC80 Protects against Aortic Dissection and Rupture by Maintaining the Contractile Smooth Muscle Cell Phenotype. Adv. Sci..

[95] Yu M., Peng J., Lu Y., Li S., Ding K. (2024). Silencing Immune-Infiltrating Biomarker CCDC80 Inhibits Malignant Characterization and Tumor Formation in Gastric Cancer. BMC Cancer.

[96] Ferraro A., Schepis F., Leone V., Federico A., Borbone E., Pallante P., Berlingieri M.T., Chiappetta G., Monaco M., Palmieri D. (2013). Tumor Suppressor Role of the CL2/DRO1/CCDC80 Gene in Thyroid Carcinogenesis. J. Clin. Endocrinol. Metab..

[97] Wang H.-H., Luo W.-Y., Lin M., Li X.-J., Xiang G.-D., Triganti S.D. (2021). Plasma Asprosin, CCDC80 and ANGPTL4 Levels Are Associated with Metabolic and Cardiovascular Risk in Patients with Inflammatory Bowel Disease. Physiol. Res..

[98] Korthagen N.M., Houtman E., Boone I., Coutinho De Almeida R., Sivasubramaniyan K., Mahdad R., Nelissen R.G.H.H., Ramos Y.F.M., Tessari M.A., Meulenbelt I. (2024). Thyroid Hormone Induces Ossification and Terminal Maturation in a Preserved OA Cartilage Biomimetic Model. Arthritis Res. Ther..

[99] Chan C.-C., Shen D., Zhou M., Ross R.J., Ding X., Zhang K., Green W.R., Tuo J. (2007). Human HtrA1 in the Archived Eyes with Age-Related Macular Degeneration. Trans. Am. Ophthalmol. Soc..

[100] Voigt A.P., Mullin N.K., Whitmore S.S., DeLuca A.P., Burnight E.R., Liu X., Tucker B.A., Scheetz T.E., Stone E.M., Mullins R.F. (2021). Human Photoreceptor Cells from Different Macular Subregions Have Distinct Transcriptional Profiles. Hum. Mol. Genet..

[101] De Luca A., De Falco M., Severino A., Campioni M., Santini D., Baldi F., Paggi M.G., Baldi A. (2003). Distribution of the Serine Protease HtrA1 in Normal Human Tissues. J. Histochem. Cytochem..

[102] Nie G.-Y., Hampton A., Li Y., Findlay J.K., Salamonsen L.A. (2003). Identification and Cloning of Two Isoforms of Human High-Temperature Requirement Factor A3 (HtrA3), Characterization of Its Genomic Structure and Comparison of Its Tissue Distribution with HtrA1 and HtrA2. Biochem. J..

[103] Zhou J., Chen F., Yan A., Xia X. (2021). Overexpression of HTRA1 Increases the Proliferation and Migration of Retinal Pigment Epithelium. Adv. Clin. Exp. Med..

[104] Shi H., Yuan M., Cai J., Lan L., Wang Y., Wang W., Zhou J., Wang B., Yu W., Dong Z. (2024). HTRA1-Driven Detachment of Type I Collagen from Endoplasmic Reticulum Contributes to Myocardial Fibrosis in Dilated Cardiomyopathy. J. Transl. Med..

[105] Tsuchiya A., Yano M., Tocharus J., Kojima H., Fukumoto M., Kawaichi M., Oka C. (2005). Expression of Mouse HtrA1 Serine Protease in Normal Bone and Cartilage and Its Upregulation in Joint Cartilage Damaged by Experimental Arthritis. Bone.

[106] Wilkinson D.J. (2022). The Serine Proteinase HtrA1 Is Ubiquitous and Abundant in Osteoarthritic Joints, but What Is It Doing?. Osteoarthr. Cartil..

[107] Oka C., Tsujimoto R., Kajikawa M., Koshiba-Takeuchi K., Ina J., Yano M., Tsuchiya A., Ueta Y., Soma A., Kanda H. (2004). HtrA1 Serine Protease Inhibits Signaling Mediated by Tgfβ Family Proteins. Development.

[108] Geyer M., Grässel S., Straub R.H., Schett G., Dinser R., Grifka J., Gay S., Neumann E., Müller-Ladner U. (2009). Differential transcriptome analysis of intraarticular lesional vs intact cartilage reveals new candidate genes in osteoarthritis pathophysiology. Osteoarthr. Cartil..

[109] Rai M.F., Sandell L.J., Barrack T.N., Cai L., Tycksen E.D., Tang S.Y., Silva M.J., Barrack R.L. (2020). A Microarray Study of Articular Cartilage in Relation to Obesity and Severity of Knee Osteoarthritis. CARTILAGE.

[110] Khan N.M., Diaz-Hernandez M.E., Martin W.N., Patel B., Chihab S., Drissi H. (2023). PH-Sensing G Protein-Coupled Orphan Receptor GPR68 Is Expressed in Human Cartilage and Correlates with Degradation of Extracellular Matrix during OA Progression. PeerJ.

[111] Di J., Chen Z., Wang Z., He T., Wu D., Weng C., Deng J., Mai L., Wang K., He L. (2023). Cartilage Tissue from Sites of Weight Bearing in Patients with Osteoarthritis Exhibits a Differential Phenotype with Distinct Chondrocytes Subests. RMD Open.

[112] Yuan Y., Xu L., Zhao Y., Yan T., Zhuo B., Bu F., Cai Q., Xu Y., Bi Q., Tong Y. (2025). Free Glutaraldehyde Gelatin Microsphere Loaded Mesenchymal Stem Cells Alleviate Osteoarthritis by Promoting Ext1 Expression. Theranostics.

[113] Winstanley-Zarach P., Rot G., Kuba S., Smagul A., Peffers M.J., Tew S.R. (2023). Analysis of RNA Polyadenylation in Healthy and Osteoarthritic Human Articular Cartilage. Int. J. Mol. Sci..

[114] Katsoula G., Steinberg J., Tuerlings M., Coutinho De Almeida R., Southam L., Swift D., Meulenbelt I., Wilkinson J.M., Zeggini E. (2022). A Molecular Map of Long Non-Coding RNA Expression, Isoform Switching and Alternative Splicing in Osteoarthritis. Hum. Mol. Genet..

[115] Han L., Cheng B., Wei W., Liu L., Cheng S., Liu H., Jia Y., Wen Y., Zhang F. (2024). Whole-Transcriptome Sequencing of Knee Joint Cartilage from Kashin–Beck Disease and Osteoarthritis Patients. Int. J. Mol. Sci..

[116] Mei L., Zhang Z., Chen R., Liu Z., Ren X., Li Z. (2023). Identification of Candidate Genes and Chemicals Associated with Osteoarthritis by Transcriptome-Wide Association Study and Chemical-Gene Interaction Analysis. Arthritis Res. Ther..

[117] Zhao S., Zhao C., Wang L., Cheng B., Zheng M., Wang X. (2025). Elucidating the Role of LGALS3BP in Coronary Atherosclerosis: Integrating Bioinformatics and Machine Learning for Advanced Insights. J. Cardiothorac. Surg..

[118] Guo X., Ji J., Jose Kumar Sreena G.S., Hou X., Luo Y., Fu X., Mei Z., Feng Z. (2020). Computational Prediction of Antiangiogenesis Synergistic Mechanisms of Total Saponins of Panax Japonicus Against Rheumatoid Arthritis. Front. Pharmacol..

[119] Wang T., He C., Hu M., Wu H., Ou S., Li Y., Fan C. (2022). Subtyping Children with Asthma by Clustering Analysis of MRNA Expression Data. Front. Genet..

[120] Yang M., Chen T., Xu Y., Liu Q., Xu X. (2024). Study on the Mechanism of Shenmai Injection in the Treatment of Sepsis. J. Cell. Mol. Med..

[121] Li C., Zheng Z. (2021). Cartilage Targets of Knee Osteoarthritis Shared by Both Genders. Int. J. Mol. Sci..

[122] Li C., Zheng Z. (2021). Males and Females Have Distinct Molecular Events in the Articular Cartilage during Knee Osteoarthritis. Int. J. Mol. Sci..

[123] Tang K., Sun L., Chen L., Feng X., Wu J., Guo H., Zheng Y. (2024). Bioinformatics Analysis and Experimental Validation of Mitochondrial Autophagy Genes in Knee Osteoarthritis. Int. J. Gen. Med..

[124] Minafra L., Bravatà V., Saporito M., Cammarata F.P., Forte G.I., Caldarella S., D’Arienzo M., Gilardi M.C., Messa C., Boniforti F. (2014). Genetic, Clinical and Radiographic Signs in Knee Osteoarthritis Susceptibility. Arthritis Res. Ther..

[125] Lin Z., Deng Z., Liu J., Lin Z., Chen S., Deng Z., Li W. (2022). Chloride Channel and Inflammation-Mediated Pathogenesis of Osteoarthritis. J. Inflamm. Res..

[126] Bengue M., Ferraris P., Baronti C., Diagne C.T., Talignani L., Wichit S., Liegeois F., Bisbal C., Nougairède A., Missé D. (2019). Mayaro Virus Infects Human Chondrocytes and Induces the Expression of Arthritis-Related Genes Associated with Joint Degradation. Viruses.

[127] Chen Y.-J., Chang W.-A., Wu L.-Y., Hsu Y.-L., Chen C.-H., Kuo P.-L. (2018). Systematic Analysis of Transcriptomic Profile of Chondrocytes in Osteoarthritic Knee Using Next-Generation Sequencing and Bioinformatics. J. Clin. Med..

[128] Steinberg J., Ritchie G.R.S., Roumeliotis T.I., Jayasuriya R.L., Clark M.J., Brooks R.A., Binch A.L.A., Shah K.M., Coyle R., Pardo M. (2017). Integrative Epigenomics, Transcriptomics and Proteomics of Patient Chondrocytes Reveal Genes and Pathways Involved in Osteoarthritis. Sci. Rep..

[129] Wu X., Liyanage C., Plan M., Stark T., McCubbin T., Barrero R.A., Batra J., Crawford R., Xiao Y., Prasadam I. (2023). Dysregulated Energy Metabolism Impairs Chondrocyte Function in Osteoarthritis. Osteoarthr. Cartil..

[130] Tang W., Li Z.-W., Miao G.-Q., Li Z.-P., Gui T., Wu C.-J., Li Z.-Y., Yang J., Zhao X.-D., Liu N. (2022). Single-Cell RNA Sequencing Reveals Transcriptional Changes in the Cartilage of Subchondral Insufficiency Fracture of the Knee. J. Inflamm. Res..

[131] Fan Y., Bian X., Meng X., Li L., Fu L., Zhang Y., Wang L., Zhang Y., Gao D., Guo X. (2024). Unveiling Inflammatory and Prehypertrophic Cell Populations as Key Contributors to Knee Cartilage Degeneration in Osteoarthritis Using Multi-Omics Data Integration. Ann. Rheum. Dis..

[132] Sun Z., Yan M., Wang J., Zhang H., Ji X., Xiao Y., Wang T., Yu T. (2024). Single-Cell RNA Sequencing Reveals Different Chondrocyte States in Femoral Cartilage between Osteoarthritis and Healthy Individuals. Front. Immunol..

[133] Sebastian A., Murugesh D.K., Mendez M.E., Hum N.R., Rios-Arce N.D., McCool J.L., Christiansen B.A., Loots G.G. (2020). Global Gene Expression Analysis Identifies Age-Related Differences in Knee Joint Transcriptome during the Development of Post-Traumatic Osteoarthritis in Mice. Int. J. Mol. Sci..

[134] Sebastian A., McCool J.L., Hum N.R., Murugesh D.K., Wilson S.P., Christiansen B.A., Loots G.G. (2021). Single-Cell RNA-Seq Reveals Transcriptomic Heterogeneity and Post-Traumatic Osteoarthritis-Associated Early Molecular Changes in Mouse Articular Chondrocytes. Cells.

[135] Wu Y., Li A., Sun N., Jiang Z., Li Y., Zhou Z., Li X., Zhao D., Leng X., Dong H. (2025). Unveiling the Mechanisms of Mechanical Loading-Induced Knee Osteoarthritis through Transcriptomics. Int. Immunopharmacol..

[136] Zhao Z., Ito A., Kuroki H., Aoyama T. (2025). Analysis of Molecular Changes and Features in Rat Knee Osteoarthritis Cartilage: Progress From Cellular Changes to Structural Damage. CARTILAGE.

[137] Hu Y., Li K., Swahn H., Ordoukhanian P., Head S.R., Natarajan P., Woods A.K., Joseph S.B., Johnson K.A., Lotz M.K. (2023). Transcriptomic Analyses of Joint Tissues during Osteoarthritis Development in a Rat Model Reveal Dysregulated Mechanotransduction and Extracellular Matrix Pathways. Osteoarthr. Cartil..

[138] Hermansson M., Sawaji Y., Bolton M., Alexander S., Wallace A., Begum S., Wait R., Saklatvala J. (2004). Proteomic Analysis of Articular Cartilage Shows Increased Type II Collagen Synthesis in Osteoarthritis and Expression of Inhibin ΒA (Activin A), a Regulatory Molecule for Chondrocytes. J. Biol. Chem..

[139] Guo D., Tan W., Wang F., Lv Z., Hu J., Lv T., Chen Q., Gu X., Wan B., Zhang Z. (2008). Proteomic Analysis of Human Articular Cartilage: Identification of Differentially Expressed Proteins in Knee Osteoarthritis. Jt. Bone Spine.

[140] Garcia B.A., Platt M.D., Born T.L., Shabanowitz J., Marcus N.A., Hunt D.F. (2006). Protein Profile of Osteoarthritic Human Articular Cartilage Using Tandem Mass Spectrometry. Rapid Commun. Mass Spectrom..

[141] Chen G., Zhang H., Bai X. (2025). FGA Can Be Used as a Promising Therapeutic Target in Osteoarthritis. BMC Musculoskelet. Disord..

[142] Steijns J.S.J.J., Green D., Peeters L.C.W., Emans P.J., Boymans T.A., Stassen R.H., Van Den Akker G.G.H., Cremers A., Jutten L.M.C., Anderson J.R. (2024). Proteomic Characterization of Regenerated Cartilage Following Knee Joint Distraction; a Human Case-Study. Connect. Tissue Res..

[143] Fan X., Wu X., Trevisan Franca De Lima L., Stehbens S., Punyadeera C., Webb R., Hamilton B., Ayyapann V., McLauchlan C., Crawford R. (2022). The Deterioration of Calcified Cartilage Integrity Reflects the Severity of Osteoarthritis—A Structural, Molecular, and Biochemical Analysis. FASEB J..

[144] Brumwell A., Raheja S., Cawley W., Birkett-Jones E., Orr S.E., Todd C., Deehan D.J., Conlon N.J., Trost M., Rice S.J. (2025). A Proteomics Investigation of Primary Human Articular Chondrocyte Isolation. Osteoarthr. Cartil. Open.

[145] Lambrecht S., Verbruggen G., Verdonk P.C.M., Elewaut D., Deforce D. (2008). Differential Proteome Analysis of Normal and Osteoarthritic Chondrocytes Reveals Distortion of Vimentin Network in Osteoarthritis. Osteoarthr. Cartil..

[146] Yu C.-J., Ko C.-J., Hsieh C.-H., Chien C.-T., Huang L.-H., Lee C.-W., Jiang C.-C. (2014). Proteomic Analysis of Osteoarthritic Chondrocyte Reveals the Hyaluronic Acid-Regulated Proteins Involved in Chondroprotective Effect under Oxidative Stress. J. Proteom..

[147] Tsolis K.C., Bei E.S., Papathanasiou I., Kostopoulou F., Gkretsi V., Kalantzaki K., Malizos K., Zervakis M., Tsezou A., Economou A. (2015). Comparative Proteomic Analysis of Hypertrophic Chondrocytes in Osteoarthritis. Clin. Proteom..

[148] Balaskas P., Goljanek-Whysall K., Clegg P.D., Fang Y., Cremers A., Smagul A., Welting T.J.M., Peffers M.J. (2023). MicroRNA Signatures in Cartilage Ageing and Osteoarthritis. Biomedicines.

[149] Destouni A., Tsolis K.C., Economou A., Papathanasiou I., Balis C., Mourmoura E., Tsezou A. (2021). Chondrocyte Protein Co-Synthesis Network Analysis Links ECM Mechanosensing to Metabolic Adaptation in Osteoarthritis. Expert Rev. Proteom..

[150] Chen Z., Tang M., Wu Z., Lin Y., Wu C., Huang H., Chen J., Zhu Z., Liu Y., Tang S. (2024). Increased Rab1a Accelerates Osteoarthritis by Inhibiting Autophagy via Activation of the MTORC1-S6K Pathway. J. Adv. Res..

[151] Li P., Che X., Gao Y., Zhang R. (2020). Proteomics and Bioinformatics Analysis of Cartilage in Post-Traumatic Osteoarthritis in a Mini-Pig Model of Anterior Cruciate Ligament Repair. Med. Sci. Monit..

[152] Fongsodsri K., Tiyasatkulkovit W., Chaisri U., Reamtong O., Adisakwattana P., Supasai S., Kanjanapruthipong T., Sukphopetch P., Aramwit P., Ampawong S. (2024). Sericin promotes chondrogenic proliferation and differentiation via glycolysis and Smad2/3 TGF-β signaling inductions and alleviates inflammation in three-dimensional models. Sci. Rep..

[153] Hart E.S., Metkar U.S., Rebello G.N., Grottkau B.E. (2009). Femoroacetabular Impingement in Adolescents and Young Adults. Orthop. Nurs..

[154] Chinzei N., Hashimoto S., Fujishiro T., Hayashi S., Kanzaki N., Uchida S., Kuroda R., Kurosaka M. (2016). Inflammation and Degeneration in Cartilage Samples from Patients with Femoroacetabular Impingement. J. Bone Jt. Surg..

[155] Philippon M.J., Schenker M.L. (2006). Arthroscopy for the Treatment of Femoroacetabular Impingement in the Athlete. Clin. Sports Med..

[156] Pascual-Garrido C., Kamenaga T., Brophy R.H., Shen J., O’Keefe R.J., Clohisy J.C. (2022). Otto Aufranc Award: Identification of Key Molecular Players in the Progression of Hip Osteoarthritis Through Transcriptomes and Epigenetics. J. Arthroplast..

[157] Kuhns B.D., Reuter J.M., Hansen V.L., Soles G.L., Jonason J.H., Ackert-Bicknell C.L., Wu C., Giordano B.D. (2023). Whole-genome RNA Sequencing Identifies Distinct Transcriptomic Profiles in Impingement Cartilage between Patients with Femoroacetabular Impingement and Hip Osteoarthritis. J. Orthop. Res..

[158] Huang J., Liu M., Zhang H., Sun G., Furey A., Rahman P., Zhai G. (2024). Multi-Omics Integrative Analyses Identified Two Endotypes of Hip Osteoarthritis. Metabolites.

[159] Huang J., Liu M., Furey A., Rahman P., Zhai G. (2025). Transcriptomic Analysis of Human Cartilage Identified Potential Therapeutic Targets for Hip Osteoarthritis. Hum. Mol. Genet..

[160] Steinberg J., Brooks R.A., Southam L., Bhatnagar S., Roumeliotis T.I., Hatzikotoulas K., Zengini E., Wilkinson J.M., Choudhary J.S., McCaskie A.W. (2018). Widespread Epigenomic, Transcriptomic and Proteomic Differences between Hip Osteophytic and Articular Chondrocytes in Osteoarthritis. Rheumatology.

[161] Aki T., Hashimoto K., Ogasawara M., Itoi E. (2018). A Whole-Genome Transcriptome Analysis of Articular Chondrocytes in Secondary Osteoarthritis of the Hip. PLoS ONE.

[162] Southan J., McHugh E., Walker H., Ismail H.M. (2020). Metabolic Signature of Articular Cartilage Following Mechanical Injury: An Integrated Transcriptomics and Metabolomics Analysis. Front. Mol. Biosci..

[163] Hosseininia S., Önnerfjord P., Dahlberg L.E. (2019). Targeted Proteomics of Hip Articular Cartilage in OA and Fracture Patients. J. Orthop. Res..

[164] Hsueh M.-F., Kraus V., Önnerfjord P. (2017). Cartilage Matrix Remodelling Differs by Disease State and Joint Type. Eur. Cells Mater..

[165] Renes J.S., Reedijk A.M.J., Losekoot M., Kant S.G., Van Der Steen M., Van Der Kaay D.C.M., Hokken-Koelega A.C.S., Van Duyvenvoorde H.A., De Bruin C. (2024). Clinical Characteristics of Pathogenic ACAN Variants and 3-Year Response to Growth Hormone Treatment: Real-World Data. Horm. Res. Paediatr..

[166] Doege K.J., Sasaki M., Kimura T., Yamada Y. (1991). Complete Coding Sequence and Deduced Primary Structure of the Human Cartilage Large Aggregating Proteoglycan, Aggrecan. Human-Specific Repeats, and Additional Alternatively Spliced Forms. J. Biol. Chem..

[167] Kiani C., Chen L., Wu Y.J., Yee A.J., Yang B.B. (2002). Structure and Function of Aggrecan. Cell Res..

[168] Dateki S. (2017). ACAN Mutations as a Cause of Familial Short Stature. Clin. Pediatr. Endocrinol..

[169] Seror J., Merkher Y., Kampf N., Collinson L., Day A.J., Maroudas A., Klein J. (2011). Articular Cartilage Proteoglycans As Boundary Lubricants: Structure and Frictional Interaction of Surface-Attached Hyaluronan and Hyaluronan–Aggrecan Complexes. Biomacromolecules.

[170] Krawetz R.J., Wu Y.E., Bertram K.L., Shonak A., Masson A.O., Ren G., Leonard C., Kapoor M., Matyas J.R., Salo P.T. (2022). Synovial Mesenchymal Progenitor Derived Aggrecan Regulates Cartilage Homeostasis and Endogenous Repair Capacity. Cell Death Dis..

[171] Bay-Jensen A.C., Mobasheri A., Thudium C.S., Kraus V.B., Karsdal M.A. (2022). Blood and Urine Biomarkers in Osteoarthritis—An Update on Cartilage Associated Type II Collagen and Aggrecan Markers. Curr. Opin. Rheumatol..

[172] Dong Y., Lin L., Ji Y., Cheng X., Zhang Z. (2023). Cabozantinib Prevents AGEs-Induced Degradation of Type 2 Collagen and Aggrecan in Human Chondrocytes. Aging.

[173] Li H., Chen J., Li B., Fang X. (2020). The Protective Effects of Dulaglutide against Advanced Glycation End Products (AGEs)-Induced Degradation of Type II Collagen and Aggrecan in Human SW1353 Chondrocytes. Chem.-Biol. Interact..

[174] Marouf B.H. (2021). Effect of Resveratrol on Serum Levels of Type II Collagen and Aggrecan in Patients with Knee Osteoarthritis: A Pilot Clinical Study. BioMed Res. Int..

[175] Kobayashi M., Kawabata K., Kusaka-Kikushima A., Sugiyama Y., Mabuchi T., Takekoshi S., Miyasaka M., Ozawa A., Sakai S. (2016). Cartilage Oligomeric Matrix Protein Increases in Photodamaged Skin. J. Investig. Dermatol..

[176] Cui J., Zhang J. (2022). Cartilage Oligomeric Matrix Protein, Diseases, and Therapeutic Opportunities. Int. J. Mol. Sci..

[177] Posey K.L., Coustry F., Hecht J.T. (2018). Cartilage Oligomeric Matrix Protein: COMPopathies and Beyond. Matrix Biol..

[178] Maly K., Andres Sastre E., Farrell E., Meurer A., Zaucke F. (2021). COMP and TSP-4: Functional Roles in Articular Cartilage and Relevance in Osteoarthritis. Int. J. Mol. Sci..

[179] Istiak A., Lawrence A., Boesel J., Kayes M.I., Bahar Moni A.S., Faisal T.R. (2025). Therapeutic Potential of COMP and TIMP-3 in Preserving Cartilage Integrity Compromised by Proteases: A Histo-Mechanical Study. J. Mech. Behav. Biomed. Mater..

[180] Bernardo B.C., Belluoccio D., Rowley L., Little C.B., Hansen U., Bateman J.F. (2011). Cartilage Intermediate Layer Protein 2 (CILP-2) Is Expressed in Articular and Meniscal Cartilage and Down-Regulated in Experimental Osteoarthritis. J. Biol. Chem..

[181] Li X., Li Y., Wang G., Fan X., Shi T., Gao F., Duan F. (2025). CHRDL2 Inhibits the Progression of Osteoarthritis by Regulating the Balance of Th17/Treg. Cytotechnology.

[182] Rivera S., Khrestchatisky M., Kaczmarek L., Rosenberg G.A., Jaworski D.M. (2010). Metzincin Proteases and Their Inhibitors: Foes or Friends in Nervous System Physiology?. J. Neurosci..

[183] Stanton H., Melrose J., Little C.B., Fosang A.J. (2011). Proteoglycan Degradation by the ADAMTS Family of Proteinases. Biochim. Biophys. Acta (BBA) -Mol. Basis Dis..

[184] De Groot R., Folgado P.B., Yamamoto K., Martin D.R., Koch C.D., Debruin D., Blagg S., Minns A.F., Bhutada S., Ahnström J. (2025). Cleavage of Cartilage Oligomeric Matrix Protein (COMP) by ADAMTS4 Generates a Neoepitope Associated with Osteoarthritis and Other Forms of Degenerative Joint Disease. Matrix Biol..

[185] Yang B., Ni J., Long H., Huang J., Yang C., Huang X. (2018). IL-1β-induced MiR-34a Up-regulation Inhibits Cyr61 to Modulate Osteoarthritis Chondrocyte Proliferation through ADAMTS-4. J. Cell. Biochem..

[186] Tew S.R., Clegg P.D., Brew C.J., Redmond C.M., Hardingham T.E. (2007). SOX9 Transduction of a Human Chondrocytic Cell Line Identifies Novel Genes Regulated in Primary Human Chondrocytes and in Osteoarthritis. Arthritis Res. Ther..

[187] Tsuritani K., Takeda J., Sakagami J., Ishii A., Eriksson T., Hara T., Ishibashi H., Koshihara Y., Yamada K., Yoneda Y. (2010). Cytokine Receptor-Like Factor 1 Is Highly Expressed in Damaged Human Knee Osteoarthritic Cartilage and Involved in Osteoarthritis Downstream of TGF-β. Calcif. Tissue Int..

[188] Xu H., Ding C., Guo C., Xiang S., Wang Y., Luo B., Xiang H. (2021). Suppression of CRLF1 Promotes the Chondrogenic Differentiation of Bone Marrow-Derived Mesenchymal Stem and Protects Cartilage Tissue from Damage in Osteoarthritis via Activation of MiR-320. Mol. Med..

[189] Yang G., Ji B., Li H., Liu X., Liu G., Sun J., Yao Y., Li Y., Liu S., Xiao W. (2025). Inhibition of CRLF1 Expression by MiR-8485 Alleviates IL-1β-Induced Chondrocyte Inflammation, Apoptosis, and Extracellular Matrix Degradation. Int. Immunopharmacol..

[190] Lu Z., Wang D., Sun Y., Dai Y. (2024). ENO1 Regulates IL-1β-Induced Chondrocyte Inflammation, Apoptosis and Matrix Degradation Possibly through the Potential Binding to CRLF1. Tissue Cell.

[191] Tang Y., Hong F., Ding S., Yang J., Zhang M., Ma Y., Zheng Q., Yang D., Jin Y., Ma C. (2023). METTL3-Mediated M6A Modification of IGFBP7-OT Promotes Osteoarthritis Progression by Regulating the DNMT1/DNMT3a-IGFBP7 Axis. Cell Rep..

[192] Yammani R.R., Carlson C.S., Bresnick A.R., Loeser R.F. (2006). Increase in Production of Matrix Metalloproteinase 13 by Human Articular Chondrocytes Due to Stimulation with S100A4: Role of the Receptor for Advanced Glycation End Products. Arthritis Rheum..

