# Supplementary material for: Omics Studies in Osteoarthritis: A Review of Molecular Mechanisms and Candidate Molecules
Source: Biomolecules. 2026 Sep 18;16(9):1363. doi: 10.3390/biom16091363 (PMC13604700; doi:10.3390/biom16091363)
Supplement: Supplementary file 1 [file biomolecules-16-01363-s001.zip › Supplemental Table S1.pdf]

Supplemental Table S1. PubMed advanced search conducted on November 30th, 2025. Six queries were used giving a total of **1259** records.

Search	Query	Items found
①	((((cartilage[Title/Abstract]) AND ((osteoarthritis[Title/Abstract]) OR (OA[Title/Abstract]))) AND (knee[Title/Abstract]) AND(hip[Title/Abstract]))) AND ((transcriptom*[Title/Abstract]) OR (RNA sequencing[Title/Abstract]) OR (RNA-Seq[Title/Abstract]) OR (Gene expression[Title/Abstract]))	40
②	((((cartilage[Title/Abstract]) AND ((osteoarthritis[Title/Abstract]) OR (OA[Title/Abstract]))) AND (knee[Title/Abstract]) AND(hip[Title/Abstract]))) AND ((proteom*[Title/Abstract]) OR (Mass spectrometry[Title/Abstract]) OR (MS[Title/Abstract]) OR (Molecular profiling[Title/Abstract]))	19
③	(((cartilage[Title/Abstract]) AND ((osteoarthritis[Title/Abstract]) OR (OA[Title/Abstract]))) AND (knee[Title/Abstract])) AND ((transcriptom*[Title/Abstract]) OR (RNA sequencing[Title/Abstract]) OR (RNA-Seq[Title/Abstract]) OR (Gene expression[Title/Abstract]))	688
④	(((cartilage[Title/Abstract]) AND ((osteoarthritis[Title/Abstract]) OR (OA[Title/Abstract]))) AND (knee[Title/Abstract])) AND ((proteom*[Title/Abstract]) OR (Mass spectrometry[Title/Abstract]) OR (MS[Title/Abstract]) OR (Molecular profiling[Title/Abstract]))	352
⑤	(((cartilage[Title/Abstract]) AND ((osteoarthritis[Title/Abstract]) OR (OA[Title/Abstract]))) AND (hip[Title/Abstract])) AND ((transcriptom*[Title/Abstract]) OR (RNA sequencing[Title/Abstract]) OR (RNA-Seq[Title/Abstract]) OR (Gene expression[Title/Abstract]))	99
⑥	(((cartilage[Title/Abstract]) AND ((osteoarthritis[Title/Abstract]) OR (OA[Title/Abstract]))) AND (hip[Title/Abstract])) AND ((transcriptom*[Title/Abstract]) OR (Mass spectrometry[Title/Abstract]) OR (MS[Title/Abstract])	61

	OR (Molecular profiling[Title/Abstract]))	
